# Prime editing of the β_1_ adrenoceptor in the brain restores physiological REM sleep in a mouse model of Alzheimer’s disease

**DOI:** 10.1038/s41467-025-65964-w

**Published:** 2025-12-09

**Authors:** Desirée Böck, Lisa Tidecks, Maria Wilhelm, Waleed ElGrawani, Sian Duss, Chiara Trevisan, Dalila Vena, Anna Maria Reuss, Lucas Kissling, Matteo Ranucci, Mattia Privitera, Lisa K. Polzer, Athena E. Economides, Eleonora I. Ioannidi, Raquel Mendes, Yanik Weber, Justine Leonardi, Jonas Mumenthaler, Elina Villiger, Romain Goutagny, Chantal Mathis, Lukas Schmidheini, Tanja Rothgangl, Sharan Janjuha, Tommaso Patriarchi, Johannes Bohacek, Yaroslav Sych, Adriano Aguzzi, Jochen Winterer, Konstantinos Kompotis, Gerald Schwank

**Affiliations:** 1 Institute of Pharmacology and Toxicology, University of Zurich, Zurich, Switzerland; 2 Neuroscience Center Zurich (ZNZ), University of Zurich and ETH Zurich, Zurich, Switzerland; 3 Laboratory of Molecular and Behavioral Neuroscience, Institute for Neuroscience, Department of Health Sciences and Technology, ETH Zurich, Zurich, Switzerland; 4 Institute of Neuropathology, University of Zurich, Zurich, Switzerland; 5 Institute of Chemistry and Biochemistry, Free University of Berlin, Berlin, Germany; 6 University of Strasbourg, CNRS, LNCA UMR 7364, Strasbourg, France; 7 Institute of Cellular and Integrative Neuroscience, CNRS UPR3212, University of Strasbourg, Strasbourg, France; 8 Laboratory of Systems Neuroscience, Institute for Neuroscience, Department of Health Sciences and Technology, ETH Zurich, Zurich, Switzerland

**Keywords:** Gene therapy, CRISPR-Cas9 genome editing, Alzheimer's disease, Sequencing

## Abstract

Prime editing offers versatile genome modifications with therapeutic potential; yet its use to modulate neural circuitry remains underexplored. Here, we used adeno-associated viral vectors to deliver prime editors into the mouse brain and introduced the naturally occurring *Adrb1*^A187V^ variant of the β1-adrenergic receptor, linked to short sleep in humans and mice. Editing reached up to 28.1% in the cortex six months after intracerebroventricular injection and increased excitability of β1-noradrenergic neurons. This enhanced wake-associated behaviors, including home cage activity, locomotion, exploration, and recognition memory, while reducing slow wave activity (SWA) during non-rapid eye movement (NREM) sleep indicating reduced build-up of sleep pressure during active phases. In a mouse model of Alzheimer’s disease, *Adrb1*^A187V^ installation restored physiological REM sleep and again reduced NREM sleep SWA following spontaneous activity. Together, these findings demonstrate the feasibility of prime editing for reprogramming genetic circuits in the brain and reveal beneficial effects of the *Adrb1*^A187V^ variant on activity and sleep regulation.

## Abstract (web summary)

This study finds that prime editing of Adrb1^A187V^ in the mouse brain boosts excitability of β1- adrenergic neurons, wake behaviors, and memory, while lowering sleep pressure. In an Alzheimer’s model, Adrb1^A187V^ also restored physiological REM sleep amounts.

## Introduction

Prime editing is a versatile genome editing technology that enables the precise introduction of a wide array of small-sized genetic changes, including transversion/transition mutations, insertions, and deletions^1^. Prime editors (PEs) consist of an *Sp*Cas9 nickase (H840A) fused to an engineered reverse transcriptase (RT) derived from the Moloney murine leukemia virus (M-MLV; hereafter referred to as PE2)^1^, and a prime editing guide RNA (pegRNA), which includes a primer binding site (PBS) and an edit-containing programmable RT template (RTT) fused to the 3’ end of the guide RNA scaffold. Unlike classical Cas9 nucleases, PEs do not rely on the induction of DNA double-strand breaks (DSB) and homology-directed repair (HDR) from donor DNA for precise editing, making prime editing particularly promising for applications in non-dividing cells such as hepatocytes, retinal cells, and neurons^2–5^. Despite its potential, the typical prime editing efficiency is substantially lower when compared to Cas9 nucleases or base editors^2,6–8^. Several studies have therefore proposed strategies to enhance prime editing rates^1,2,9–13^. While these approaches facilitated the installation of edits in numerous cell line studies and advanced our understanding of the cellular mechanisms affecting prime editing outcomes, there are still only a few reports of successful in vivo prime editing in animals^2–5,14,15^. Furthermore, the potential of prime editing to modulate genetic circuits within the central nervous system (CNS) is yet to be explored.

In this study, we developed an adeno-associated virus (AAV)-mediated prime editing approach to introduce the naturally occurring *Adrb1*^A187V^ variant of the β1-adrenoceptor (β1-AR) in the murine brain. β-ARs are activated by the neurotransmitter norepinephrine (NE; schematic representation in supplementary fig. 1a)^16^ and are involved in the regulation of a wide range of brain functions, including sleep and wakefulness^17^, memory^18^, and stress- and anxiety-related responses^19,20^. β1-ARs are particularly important for the physiological functioning of the sympathetic nervous system^21,22^, with the autosomal dominant *Adrb1*^A187V^ variant being linked to short sleep in humans and mice^23^. Additionally, *Adrb1*^A187V^ has been shown to restore REM sleep disturbances and alleviate tau accumulation in a mouse model of tauopathy^24^.

By employing optimized AAV vectors and pegRNA designs, we were able to introduce the *Adrb1*^A187V^ variant into healthy mice with an efficiency of up to 28.1% in whole cortex tissue. This resulted in increased locomotor activity, exploration, recognition memory, and neuronal excitability as well as decreased NREM sleep EEG delta power and SWA (1.0–4.5 Hz). Furthermore, when *Adrb1*^A187V^ was introduced into a mouse model of Alzheimer’s disease (AD), it restored physiological REM sleep amounts during the quiescent period and again decreased NREM sleep EEG delta power and SWA, particularly during the dark period where mice are mostly active. Taken together, our data suggest that installation of the *Adrb1*^A187V^ variant in the CNS via prime editing can have beneficial effects on various behaviors, inlcuding locomotion, exploration, memory, or sleep, under physiological and pathological conditions.

## Results

### Installation of the *Adrb1*^A187V^ mutation via prime editing in cell lines

**Fig. 1:**
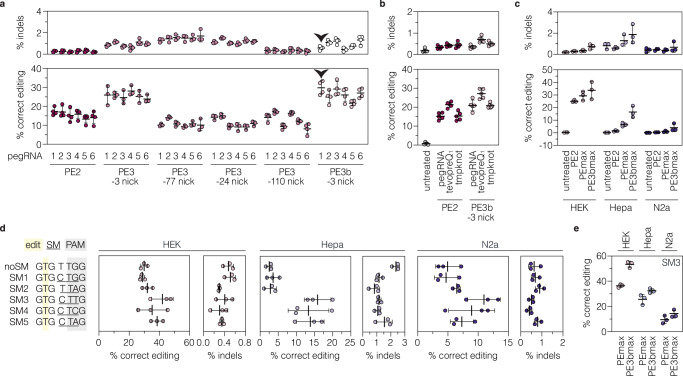
Optimization of pegRNAs and ngRNAs for the *Adbr1* locus. **a** Editing and indel rates of six pegRNAs alone (PE2, *n* = 4) or combined with different ngRNAs (PE3 or PE3b; *n *= 3 or 4 per group) in HEK cells with the integrated *Adrb1* target site. The location of the second nick relative to the installed edit is indicated below the prime editing approach. The black arrowhead labels pegRNA1 and the PE3b ngRNA, which displayed high on-target and low indel performance. **b** Editing and indel rates of tevopreQ_1_- and tmpknot-epegRNA1 in HEK cells with the integrated *Adrb1* target site (*n* = 5 per group). **c** Comparison of editing and indel rates (*n* = 3 per group) of the original PE2 or the optimized PEmax variant and epegRNA1 without (PEmax) or with a ngRNA (PE3bmax). **d** Editing and indel rates of epegRNA1 encoding different SMs (*n* = 3 per group). The position of the edit (yellow), SMs (underlined), and PAM sequence (gray) are indicated. **e** Editing and indel rates (*n* = 3 per group) of epegRNA-SM3 without (PEmax) and with a ngRNA (PE3bmax). Data are displayed as means±s.d. of biological replicates. Each datapoint represents one independent experiment. SM, silent mutation; PAM, protospacer adjacent motif. Source data are provided as a Source Data file.

To develop a prime editing approach for efficient installation of the p.A187V mutation in the *Adrb1* gene, we first tested different pegRNA designs at the endogenous locus in murine cell lines. We designed six pegRNAs encoding the respective C-to-T edit with varying lengths of the RTT (11, 13, or 15 nucleotides [nt]) and PBS (10 or 13nt; supplementary fig. 1b). Vectors expressing the pegRNAs were co-delivered with a PE2 expressing plasmid into murine Hepa1-6 and Neuro2a cell lines (hereafter referred to as Hepa and N2a). However, deep sequencing of the target locus revealed low editing rates with all tested pegRNAs ( < 2%; supplementary fig. 1c), possibly due to chromatin repression of the *Adrb1* locus in these cell lines^25,26^ (Supplementary fig. 1d). We therefore generated a HEK293T (hereafter referred to as HEK) cell line where the targeted *Adrb1* sequence was integrated into the genome using the PiggyBac transposon system^27^. Transfection of PE2 together with the different pegRNAs—either alone or in combination with different nicking sgRNAs (ngRNAs) that cut the non-edited strand simultaneously to (PE3) or after resolution of the edited strand (PE3b)^1,2,9,10^—identified pegRNA1 with a 10-nt PBS and an 11-nt RTT as the most efficient pegRNA for installing the *Adrb1*^A187V^ mutation without inducing high levels of indels (Fig. 1a; supplementary fig. 1b, e). Adding the stabilizing structural motif tevopreQ_1_^10^ to the 3’ end of pegRNA1 further enhanced its activity by 1.4-fold (Fig. 1b), while adding stabilizing motifs to the 3’ end of ngRNAs did not alter prime editing efficiencies (Supplementary fig. 2). Based on these results, we used the tevopreQ_1_-modified pegRNA1 (epegRNA1) for subsequent experiments.

DNA mismatch repair (MMR) has been shown to impede prime editing activity and promote undesired indel byproducts^9,28^. Since the CNS contains MMR proficient cells^29^, we next integrated the *Adrb1* target site into Hepa and N2a cells, which, in contrast to HEK cells, are not deficient in MMR. As expected, epegRNA1 in combination with PE2 or a codon-optimized PE variant that harbors additional mutations in the Cas9 nickase domain (R221K, N394K) and an improved linker/NLS design (termed PEmax), resulted in markedly lower editing efficiency in these cell lines compared to HEK cells (Hepa, 1.5 ± 0.5% for PE2 and 6.5 ± 1.5% for PEmax; N2a, 0.4 ± 0.1% for PE2 and 1.1±0.6% for PEmax; Fig. 1c). These results prompted us to assess if the inhibitory effect of MMR on prime editing could be circumvented by i) using a PE3b ngRNA, or ii) modifying epegRNA1 to additionally introduce silent mutations (SMs) in the protospacer and PAM^9,30^. Co-transfection of a PE3b ngRNA indeed led to a pronounced increase of editing rates in Hepa (2.5-fold) and N2a cells (3.9-fold; Fig.1c). Similarly, three of the five epegRNA1 variants encoding for SMs in the protospacer and/or PAM led to substantially higher editing in Hepa (up to 5.2-fold; Fig. 1d) and N2a cells (up to 2.3-fold; Fig. 1d), with epegRNA1-SM3 showing highest editing efficiencies (HEK, 44.0 ± 3.7%; Hepa, 15.9 ± 3.6%; N2a, 11.0 ± 2.7%; Fig. 1d). Moreover, combining epegRNA1-SM3 with a PE3b ngRNA further enhanced editing rates (1.5-fold for HEK; 1.3-fold for Hepa; 1.5-fold for N2a; Fig. 1e).

### Generation of intein-split PE vectors for AAV-mediated prime editing in the brain

Due to low immunogenicity, rare genomic integration, and the ability to efficiently cross the blood-brain-barrier, AAV-PHP.eB is a promising vector for in vivo delivery of PEs into the CNS^31^. As PEs exceed the packaging limit of AAVs ( ~ 5 kb including ITRs)^32^, we and others have previously employed the *Npu* intein-mediated protein trans-splicing system to split PEs into two parts for expression from two separate AAVs^3,5^.

**Fig. 2:**
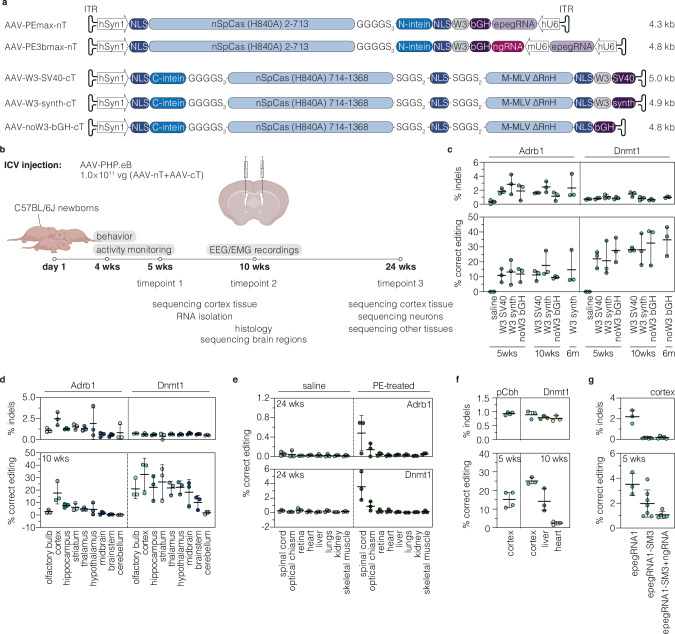
In vivo prime editing at the *Dnmt1* and *Adrb1* locus in the brain. **a** Schematic representation of AAV vector designs used in vivo and their corresponding lengths in kilobases (kb, including ITRs) for neuron-specific expression of PEmax or PE3bmax. Constructs are not depicted to scale. **b** Schematic representation of the experimental setup and timeline. Created in BioRender (https://BioRender.com/uq1jtiz). **c** In vivo prime editing and indel rates of different AAV vector designs in mice cortices at 5 weeks, 10 weeks, and 6 months post-injection (*n *= 3 mice per group). **d** Editing and indel rates at the *Adrb1* (AAV-PE3bmax-nT and AAV-W3-synth-cT) and *Dnmt1* (AAV-PEmax-nT and AAV-noW3-bGH-cT) locus in different brain regions at 10 weeks post-injection (*n* = 3 mice per group). **e** Frequency of *Adrb1* and *Dnmt1* edits in other tissues in saline- and PE-treated animals at 24 weeks post-injection (*n* = 3 mice per group). Animals were treated with the same AAV preparations as in (**d**). Skeletal muscle tissue was isolated from the quadriceps femoris. **f** Editing rates at the *Dnmt1* locus in mice cortices (5 and 10 weeks post-injection), liver (10 weeks post-injection), and heart (10 weeks post-injection) after ICV injection (*n* = 3 or 4 mice per group). Animals were treated with AAV-PEmax-noW3-synth-nT and AAV-noW3-synth-cT under the control of the ubiquitous Cbh promoter^38^. **g** Comparison of editing and indel rates at the *Adrb1* locus in the cortex for PEmax complexed with epegRNA1 (*n* = 3 mice) or epegRNA1-SM3 (with and without a PE3bmax ngRNA, *n* = 7 or 5 mice) at 5 weeks post-injection. Animals were treated with AAV-PEmax-nT or AAV-PE3bmax-nT and AAV-W3-synth-cT. Data are displayed as means±s.d. with each datapoint representing an animal. Each datapoint represents one animal. ITR, inverted terminal repeat; nT/cT, N-/C-terminal PEmax AAV vector; phSyn1, human synapsin 1 promoter; NLS, nuclear localization signal; n*Sp*Cas9, *Sp*Cas9 nickase; M-MLV, Moloney Murine Leukemia virus; W3, woodchuck hepatitis virus post-transcriptional regulatory element; hU6/mU6, human/mouse U6 promoter; SV40, Simian virus 40; pA, polyA signal; synth, synthetic polyA signal; bGH, bovine growth hormone polyA signal; kbp, kilobasepairs; vg, vector genomes; wks, weeks; m, months; SM, silent mutation; pCbh, truncated chimeric CMV/chicken-β-actin hybrid promoter; EEG, electroencephalography; EMG, electromyography. Source data are provided as a Source Data file.

To further optimize intein-split PE vectors towards accommodating a variety of promoters and terminators, we generated nine PE variants split at different surface-exposed sites and assessed their activity on a GFP reporter locus and the *Adrb1* target site in cell lines. Of the tested variants PE-p.713/714 was the most efficient, maintaining over 85% of the activity of unsplit PE (Supplementary fig. 3a, b). Next, we generated AAV expression cassettes where the N- and C-terminal fragments of PE-p.713/714 were cloned downstream of the neuron-specific human synapsin 1 (hSyn1) promoter^33^. While the vector encoding the N-terminal PE fragment was small enough to accommodate the commonly used W3-bGH terminator^34,35^ and U6 expression cassettes for the epegRNA1 and PE3b ngRNA (Fig. 2a), the C-terminal fragment would have exceeded the AAV packaging limit in the classical PE expression vector design (Supplementary fig. 3c). Therefore, we generated C-terminal constructs where we removed the dispensable RNaseH domain from the RT^3,36^ (Supplementary fig. 3c, d) and exchanged the W3-bGH terminator with smaller terminators (Fig. 2a), which either lacked the W3 post-transcriptional regulatory element or employed shorter polyadenylation signals (SV40 or synthetic polyA instead of bGH polyA). When tested in cell lines, none of the shorter terminators resulted in lower editing rates compared to the W3-bGH terminator, with the bGH terminator lacking the W3 element leading to slightly higher editing rates compared to the terminators containing shorter polyA signals (Supplementary fig. 3e).

To setup AAV prime editing approaches in the brain, we next packaged the N- and C-terminal PE expression vectors into AAV-PHP.eB particles (Fig. 2a, b). In addition to targeting the *Adrb1* locus with epegRNA1 in the PE3b approach, we also treated mice with a previously validated pegRNA installing a G-to-C mutation at the *Dnmt1* locus^3^ (Supplementary table 1; serves as a positive control), which acts redundantly with *Dnmt3a* and does not lead to phenotypes when inactivated in the CNS^37^. After confirming efficient packaging of the N- and C-terminal PE expression vectors into AAV-PHP.eB capsids by negative staining electron microscopy (Supplementary fig. 4a–d), particles were delivered to the lateral ventricles of newborn mice via intracerebroventricular (ICV) injection at a dose of 5 × 10^10^ vector genomes (vg) per construct and animal (Fig. 2b). At 5 weeks post-injection, mice cortices showed on average 13.3 ± 8.3% editing at the *Adrb1* locus (for AAV-W3-synth-cT; see Fig. 2a for vector details) and 27.7 ± 8.2% editing at the *Dnmt1* locus (for AAV-noW3-bGH-cT; see Fig. 2a for vector details), without significant differences between the three terminators utilized in the C-terminal PE vectors (Fig. 2c). In line with these results, RT-qPCR analysis revealed similar C-terminal PE expression levels from the different vectors (Supplementary fig. 4e, f). Editing rates were also maintained over time as assessed by deep amplicon sequencing at 10 weeks (*Adrb1*, 17.6 ± 8.6% for AAV-W3-synth-cT; *Dnmt1*, 32.7 ± 13.1% for AAV-noW3-bGH-cT) and 6 months post-injection (*Adrb1*, 14.7 ± 11.6% for AAV-W3-synth-cT; *Dnmt1*, 34.8 ± 9.8% for AAV-noW3-bGH-cT; Fig. 2c). Similarly, the formation of indels, primarily caused by the nickase activity of *Sp*Cas9 rather than integrations of the pegRNA scaffold (Supplementary fig. 5), did not increase over time and remained at 2.3 ± 1.8% at the *Adrb1* locus and 1.0 ± 0.1% at the *Dnmt1* locus (cortices at 6 months; Fig. 2c).

When we next assessed variations in editing rates across distinct brain regions, we found that editing efficiencies roughly reflected the biodistribution of AAV-PHP.eB in the brain after ICV injection (Fig. 2d; supplementary fig. 6)^38^. As expected, the use of the hSyn1 promoter also limited PE expression to neurons (Supplementary fig. 7), resulting in 2- (*Dnmt1*) or 4-fold (*Adrb1*) increased editing rates in neuron-enriched cell populations instead of whole cortex tissue (Supplementary fig. 8). Further confirming neuron-specific prime editing, we did not observe editing above background levels in any of the other tested organs except the spinal cord (Fig. 2e), whereas exchanging the hSyn1 promoter to the ubiquitously active Cbh promoter^39^ led to substantial editing at the *Dnmt1* locus in the brain (25.0 ± 1.9%; Fig. 2f), liver (14.2 ± 6.2%; Fig. 2f), and heart (2.5 ± 0.9%; Fig. 2f) at 10 weeks post-ICV injection.

Finally, we also assessed the activity of epegRNA1-SM3 (with or without the PE3bmax nicking sgRNA), which resulted in higher on-target editing and lower indel formation than epegRNA1 in Hepa and N2a cells (Fig. 1d). However, while indel rates were significantly reduced with epegRNA1-SM3 and comparable to the levels of saline-treated controls (Fig. 2g), editing rates were lower than with epegRNA1, without (2.1 ± 1.3% vs. 3.5 ± 0.9%; Fig. 2g) or in combination with the PE3b ngRNA (1.3 ± 0.5%, Fig. 2g; vs. 13.2 ± 8.3%, Fig. 2c). Notably, as AAV biodistribution also varies within isolated brain structures (Supplementary fig. 6a), whole-region prime editing efficiencies may not accurately recapitulate editing rates across substructures or neuronal subpopulations (Supplementary fig. 9).

### Installing *Adrb1*^A187V^ alters neuronal excitability and EEG slow wave spectral power during NREM sleep

**Fig. 3:**
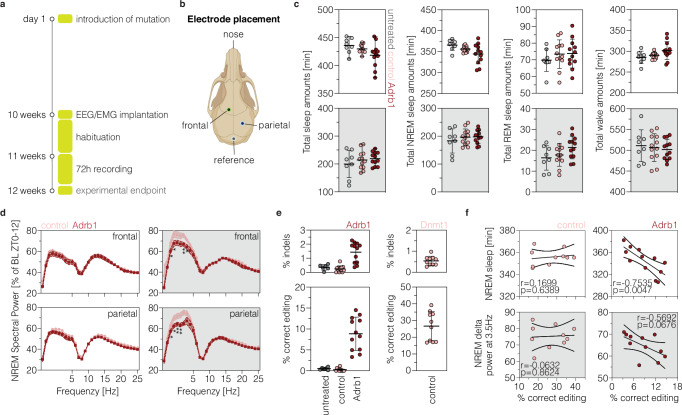
Installing the *Adrb1*^A187V^ variant alters EEG slow wave spectral power during NREM sleep. **a** Experimental setup and timeline of EEG/EMG experiments starting at postnatal day 1. **b** Schematic depiction of EEG electrode placements on the frontal and parietal lobes. Created in BioRender (https://BioRender.com/tb725y3). **c** Quantifications of total time spent asleep or in NREM sleep, REM sleep, and wakefulness. Averages over the recording days are shown (untreated, *n* = 8 mice; control and Adrb1, *n* = 12 mice per group). **d** Normalized NREM sleep EEG spectral power in the 12 h light (left) and dark phase (right; frontal: *P = 0.0217; *P = 0.0469; **P = 0.0037; *P = 0.0237; parietal: *P = 0.0287; *P = 0.0469; **P = 0.0071; **P = 0.0018) as a percentage of the total power in all vigilance states (*n* = 12 mice per group). **e** Editing and indel rates in untreated (*n* = 6 mice), control- (*n* = 10 mice) and Adrb1-treated animals (*n* = 12 mice) at 12 weeks post-injection. **f** Pearson correlations of on-target prime editing frequency at the *Dnmt1* (left, *n* = 10 mice) or *Adrb1* (right, *n* = 12 mice) locus in whole-cortex tissues and the total amount of NREM sleep (left) during the light phase or normalized NREM sleep EEG delta power at 3.5 Hertz (Hz; right) during the dark phase. Correlation coefficients, *P*-values, and 95% confidence intervals are indicated in the respective plots. Data are displayed as means±s.e.m. (**c**, **d**) or means±s.d. (e) and were analyzed using an two-way ANOVA with Tukey’s multiple comparisons test (c) or a mixed model two-way ANOVA with Geisser Greenhouse correction followed by Šidák’s multiple comparisons test (d). If not indicated, differences are not statistically significant (*P* > 0.05). Areas highlighted in white indicate the light phase (ZT0-12) and areas highlighted in gray indicate the dark phase (ZT12-24). Control-treated animals were treated with PEmax and an epegRNA targeting *Dnmt1*. Adrb1-treated animals were injected with PE3bmax and epegRNA1 targeting *Adrb1*. Untreated age-matched healthy mice were used as additional controls (untreated). Each data point represent one animal (**c**, **e**, **f**). ZT, zeitgeber time; min, minute; h, hour; REM, rapid eye movement; NREM, non-rapid eye movement; Hz, Hertz; EEG, electroencephalography; EMG, electromyography.

Since *Adrb1*^A187V^ is a naturally occurring variant associated with increased neuronal excitability and short sleep in humans and mice^23^, we next examined whether introducing this variant via prime editing modulates 1) Adrb1 receptor function using local field potential (LFP) recordings (Supplementary fig. 10a), and 2) sleep/wake patterns using simultaneous electroencephalography (EEG)/electromyography (EMG) recordings (Fig. 3a, b). AAV-PHP.eB vectors, introducing the *Adrb1*^A187V^ variant with epegRNA1 and the PE3b ngRNA (Adrb1-treated, AAV-PE3bmax-nT and AAV-W3-synth-cT; see Fig. 2a for vector details), or control vectors, targeting the *Dnmt1* locus (control-treated, AAV-PEmax-nT and AAV-noW3-bGH-cT; see Fig. 2a for vector details), were delivered to the lateral ventricles of newborn mice via ICV injection (Supplementary fig. 10a; Fig. 3a). Untreated age-matched healthy mice were included as additional controls for EEG/EMG recordings (referred to as untreated).

At 9-11 weeks post-injection, brain slices were prepared and neuronal activity within the CA1 area of the hippocampus was recorded in the presence of the selective β1-agonist dobutamine (10 μM; supplementary fig. 10a). Our data revealed that basal synaptic transmission in the CA1 area was altered in Adrb1-treated mice (Supplementary fig. 10b). In fact, we observed a significantly increased field excitatory postsynaptic potential (fEPSP) slope at 0.3 mV afferent fiber volley amplitude, indicative of increased excitability in prime-edited neurons. Notably, deep amplicon sequencing confirmed on-target editing in all animals (Supplementary fig. 10c) and further indicated a more pronounced increase in neuronal excitability at higher prime editing efficiencies (Supplementary fig. 10c). Hence, our results are in line with previous observations reporting a reduced rheobase and an increased firing frequency in *Adrb1*^*+/*A187V^ neurons in the dorsal pons^23^.

To evaluate the effect of the variant on sleep/wake patterns, we continuously recorded EEG/EMG signals at 10 weeks post-injection for three days (Supplementary fig. 11a) where the animals were undisturbed with *ad libitum* food and water under a regular 12:12 light/dark cycle (Fig. 3a). Analysis of EEG/EMG signals revealed a robust distribution of sleep/wake cycles across the day, with on average 65.7% (Adrb1-treated), 67.4% (control-treated), or 68.5% (untreated) of sleep occurring during the light phase (Fig. 3c; supplementary fig. 11b). Mice slept on average 418.4 ± 6.2 min (Adrb1-treated), 429.7 ± 2.1 min (control-treated), or 435.9 ± 4.8 min (untreated) in the light phase and 219.1 ± 7.0 min (Adrb1-treated), 214.7 ± 9.7 min (control-treated), or 199.8 ± 17.3 min (untreated) in the dark phase (Fig. 3c), with no significant differences in the time spent in different vigilance states (Fig. 3c; supplementary fig. 11b). Subsequent power spectral analyses of EEG signals revealed no changes during time spent in REM sleep for Adrb1-treated mice (Supplementary fig. 11c). In contrast, NREM sleep exhibited a consistent reduction in EEG delta power in Adrb1-treated mice in the dark phase (between 1.5-4.5 Hz; Fig. 3d; supplementary fig. 11d). Delta power during NREM sleep, also referred to as SWA, is a well-established EEG correlate of sleep homeostasis across species^40,41^, with SWA building up during periods of wakefulness, reaching its peak at the onset of sleep, and monotonically dissipating across sleep episodes^42,43^. Reduced SWA might therefore indicate that Adrb1-treated mice accumulate less sleep pressure during periods of wakefulness in the dark or active phase (Supplementary fig. 11d). In line with this hypothesis, we observed a trend towards reduced NREM sleep in the light phase (Fig. 3c), which was more pronounced in mice with higher prime editing efficiencies (r = −0.7535, *p* = 0.0047; Fig. 3e, f). Similarly, SWA was slightly more reduced in animals with higher prime editing rates (r = −0.5692, *p* = 0.0676; Fig. 3f). Off-note, brain tissue concentrations of noradrenaline and its metabolite 3-methoxy-4-hydroxyphenylglycol (MHPG) were not affected (Supplementary fig. 12).

Taken together, installation of the *Adrb1*^A187V^ variant via prime editing modulated activity patterns of the networks involved in the regulation of sleep SWA. However, possibly due to low editing rates in certain *Adrb1*-expressing brain regions (Supplementary fig. 13), we did not observe significant changes in sleep amounts.

### Introducing the *Adrb1*^A187V^ variant modulates locomotion, exploration, and recognition memory

Apart from short sleep, humans with Familial Natural Short Sleep (FNSS) mutations commonly display greater physical activity, pain tolerance, or stress resilience^44^. We therefore assessed if installing the *Adrb1*^A187V^ variant in the murine brain also affected other behaviors.

**Fig. 4:**
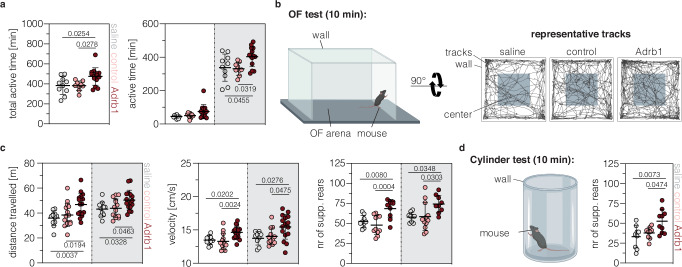
Introducing the *Adrb1*^A187V^ variant modulates locomotion and exploration. **a** General activity (expressed as total active time) in the home cage, assessed using IR sensors, in the light and dark phase (saline, *n* = 10 mice; control, *n* = 9 mice; Adrb1, *n* = 15 mice). **b** Schematic representation of the OF test and representative tracks of saline-, control-, and Adrb1-treated mice. **c** Locomotor activity (left), velocity (middle), and wall-supported rears (right) in the OF during the light and dark phase (saline, *n* = 10 mice; control, *n* = 14 mice; Adrb1, *n* = 17 mice). **d** Schematic representation (left) and quantification (right) of wall-supported rears in an unfamiliar cylinder (saline, *n* = 9 mice; control, *n* = 9 mice; Adrb1, *n* = 10 mice). The duration of each test is indicated in brackets (**b**, **d**). Areas highlighted in white indicate the light phase (ZT0-12) and areas highlighted in gray indicate the dark phase (ZT12-24). Control animals were treated with PEmax and an epegRNA targeting *Dnmt1*. Adrb1-treated animals were injected with PE3bmax and epegRNA1 targeting *Adrb1*. Data are displayed as means±s.d. and were analyzed using a two-way ANOVA with Tukey’s multiple comparisons test. If not indicated, differences were not statistically significant (*P* > 0.05). Each data point represents one animal. Schematic representations in panels (**b**) and (**d**) were created in BioRender (https://BioRender.com/w6phuta). min, minute; OF, open field; m, meter; cm, centimeter; s, second. Source data are provided as a Source Data file.

First, we monitored general activity of mice in their home cage using infrared (IR) sensors 4 weeks after AAV-PHP.eB particles (AAV-PE3bmax-nT and AAV-W3-synth-cT; see Fig. 2a for vector details) were delivered into newborn mice via ICV injection. In line with previous reports^23,44^, Adrb1-treated animals displayed longer active periods in the home cage, with a significant increase compared to age-matched saline- or control-treated animals (AAV-PEmax-nT and AAV-noW3-bGH-cT; see Fig. 2a for vector details) during the dark phase (Fig. 4a). Next, we assessed locomotor activity in the open field (OF) test (Fig. 4b). Our results show that Adrb1-treated mice covered significantly more distance (light phase: Adrb1, 46.9 ± 9.0 m; control, 38.5 ± 9.4 m; saline, 36.0 ± 5.8 m; dark phase: Adrb1, 50.4 ± 7.9 m; control, 43.9 ± 7.5 m; saline, 43.2 ± 5.6 m; Fig. 4c) and moved at higher velocity than saline- or control-treated animals (light phase: Adrb1, 14.6 ± 0.9 cm/s; control, 13.3 ± 1.2 cm/s; saline, 13.5 ± 0.7 cm/s; dark phase: Adrb1, 15.5 ± 1.8 cm/s; control, 14.0 ± 1.3 cm/s; saline, 13.8 ± 1.0 cm/s; Fig. 4c). We further observed an increase in the frequency of wall-supported rears, both in the OF (Fig. 4c) and when animals were placed in a cylinder as a novel environment (Fig. 4d), indicating enhanced exploratory or escape behavior in Adrb1-treated animals^45^. Notably, it is unlikely that the observed increase in rearing behavior is related to obsessive-compulsive-like behaviors as we did not detect differences between saline-, control-, or Adrb1-treated mice when assessing self-grooming in the OF^46^ or marble burying^47^ (Supplementary Fig. 14a, b). Moreover, while analysis of stress- or anxiety-related responses in the OF^48,49^ did not reveal significant differences between groups (Supplementary fig. 14c), Adrb1-treated mice spent significantly more time in the light compartment during the light-dark (LD) transition test^50^ (Supplementary fig. 14d). Hence, these findings support our hypothesis that Adrb1-treated mice exhibit an increased exploratory drive rather than escape, anxiety- or obsessive-compulsive-related behaviors.

Since β-ARs are also important for memory^18^, we next assessed recognition memory using the novel object (NO) recognition test (Supplementary fig. 15a). Adrb1-treated mice approached the NO more often (after 5 min: Adrb1, 61.6 ± 7.3%; control, 52.0 ± 9.0%; saline, 52.2 ± 7.4%; supplementary fig. 15b) and also spent significantly more time with it (after 5 min: Adrb1, 72.9 ± 15.2%; control, 58.2 ± 11.1%; saline, 57.2 ± 7.8%; supplementary fig. 15b). While we cannot exclude that this observation is influenced by increased exploration in Adrb1-treated mice (Fig. 4c, d), results from the NO recognition test support that installation of the *Adrb1*^A187V^ variant may have a beneficial impact on learning and memory.

While our data strongly indicate that the observed behavioral phenotypes are related to the installation of the *Adrb1*^A187V^ mutation, they could also be a consequence of i) indel formation at the targeted site, ii) editing at off-target sites of epegRNA1, or iii) tissue damage caused by AAV toxicity.

Strengthening our hypothesis that behavioral phenotypes are caused by the introduction of the *Adrb1*^A187V^ variant, we found that indel rates at the nicking site were ~16-fold lower than on-target editing rates (0.5±0.1% vs. 7.8±3.6%; supplementary fig. 16a), and that locomotor activity in the OF correlated with on-target editing rates (r = 0.7599, *p* = 0.0016) but not indel rates (r = 0.3470, *p* = 0.2242; supplementary fig. 16b). Additionally, when repeating the OF test with animals treated with PEmax and epegRNA-SM3, which is less efficient in installing *Adrb1*^A187V^, but does not induce indels above background levels (Supplementary fig. 16c), we again observed a significant increase in locomotor activity (43.9 ± 6.0 m vs. 31.2 ± 5.7 m; supplementary fig. 16d), with a direct correlation between the traveled distance and *Adrb1*^A187V^ on-target editing (r = 0.7171, *p* = 0.0697), but not indel rates (r = −0.3782, *p* = 0.4029; supplementary fig. 16e). Finally, deep sequencing of the top 5 off-target sites of epegRNA1 (identified by CHANGE-seq^51^) in cortices of treated mice revealed no editing above background levels at these locations (Supplementary fig. 17), and immunofluorescence analyses of the pan-neuronal marker NEUN and the astrocyte-specific marker GFAP did not reveal signs of neuronal death or astrogliosis in areas surrounding the injection site (Supplementary fig. 18). Our data therefore indicate that the installation of the *Adrb1*^A187V^ mutation led to the observed behavioral phenotypes and functional changes at the receptor level.

### Installing *Adrb1*^A187V^ in different brain regions recapitulates increased home cage activity

Next, we investigated which brain regions might be linked to the changes in animal behavior and separately quantified editing rates in regions where *Adrb1* is expressed^23,52–54^ (Supplementary fig. 13a,b). We found that editing rates were higher in the medial prefrontal cortex (mPFC; 12.2 ± 4.5%) and the hippocampus (HC; dorsal: 7.0±1.9%, ventral: 3.5 ± 1.3%) compared to other areas with *Adrb1* expression, including the lateral septal nucleus (1.8±1.0%), caudate putamen (3.6±1.4%), amygdala (1.3±1.2%), thalamus (3.0 ± 0.9%), paraventricular nucleus (0.6±0.3%), midbrain (1.5±1.4%), dorsal pons (0.4 ± 0.2%), and medulla oblongata (0.4±0.4%) (Supplementary fig. 13c,d). We therefore assessed if installing *Adrb1*^A187V^ directly in the mPFC or dorsal HC (dHC) via bilateral intracranial AAV injections in adult C57BL/6JRj mice could lead to similar phenotypic outcomes (Supplementary fig. 19a). Deep sequencing of whole mPFC and dHC tissues confirmed editing rates of 13.5±10.4% and 7.7±6.3%, respectively (Supplementary fig. 19b,c). Importantly, these rates are likely underestimating prime editing efficiencies in noradrenergic neurons, since isolated tissues also included glial cells and other neuronal subpopulations. While the active time in the home cage was significantly increased for Adrb1- vs. control-treated animals in the dark phase (mPFC, 386.8 ± 43.1 min vs. 323.4 ± 46.9 min; dHC, 370.9±76.2 min vs. 306.3±48.8 min; supplementary fig. 19d), locomotor activity, velocity, and the number of wall-supported rears were comparable between both groups (Supplementary fig. 19e). Likewise, we did not observe differences in obsessive compulsive-like or anxiety-related behaviors (Supplementary fig. 19f–i) or recognition memory (Supplementary fig. 19j). Thus, our data suggest that the introduction of the *Adrb1*^A187V^ variant into mPFC or dHC neurons is insufficient to globally modulate the noradrenergic network, and that multiple brain areas with *Adrb1* expression may contribute to the complex behavioral changes observed in ICV-treated mice (Fig. 4; supplementary figs. 14 and 15).

### *Adrb1*^A187V^ restores physiological REM sleep amounts and modulates NREM sleep EEG delta power in a mouse model of Alzheimer’s disease (AD)

Although chronic sleep disturbances are among the first clinical manifestations of AD^55^ and prolonged sleep deprivation is linked to neurodegeneration and cognitive decline^23^, individuals with short sleep mutations do not have an increased risk of developing these diseases. In fact, it has recently been demonstrated that FNSS alleles confer resilience against progression of tau pathology or amyloid plaque formation, or can alleviate REM sleep deficits in mice^56^. These observations prompted us to investigate whether PE-mediated introduction of the *Adrb1*^A187V^ variant could similarly improve pathologies in a mouse model of AD.

**Fig. 5:**
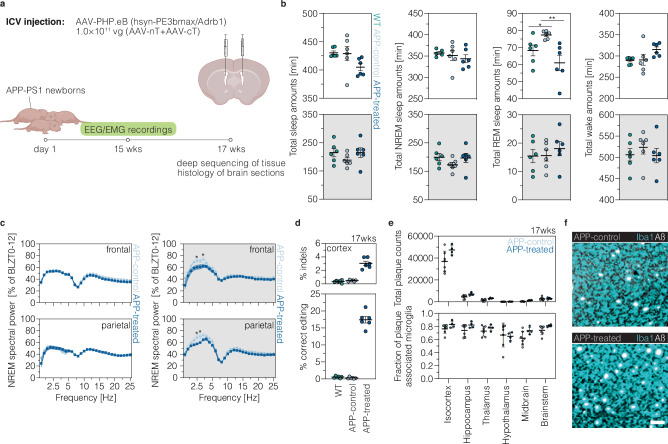
*Adrb1*^A187V^ restores physiological REM sleep amounts and modulates NREM sleep EEG delta power in a mouse model of AD. **a** Schematic representation of the experimental timeline and setup. Created in BioRender (https://BioRender.com/tb725y3). **b** Quantifications of time spent asleep or in NREM sleep, REM sleep (*P = 0.0372; **P = 0.0010), and wakefulness (*n* = 6 mice per group). Averages over the recording days are shown. **c**) Normalized NREM sleep EEG spectral power in the 12 h light (left) and dark phase (right; frontal: *P = 0.0465; *P = 0.0216; parietal: **P = 0.0088; **P = 0.0087) as a percentage of the total power in all vigilance states (*n* = 6 mice per group). **d** Editing and indel rates in WT, APP-control, and APP-treated animals at 17 wks post-injection (*n *= 6 mice per group). e,f Quantifications (**e**; APP-control, *n* = 6 mice; APP-treated, *n* = 4 mice) and representative images (**f**) of β-amyloid plaques (**e**, top) and plaques engulfed by microglia (**e**, bottom) at 17 wks post-ICV injection. Scale bar, 50 μm (**f**). Data are displayed as means±s.e.m. (**a**–**d**) or means ±s.d. (**e**) and were analyzed using a two-way ANOVA with Tukey’s multiple comparisons test (**b**) or a mixed model two-way ANOVA with Geisser Greenhouse correction followed by Šidák’s multiple comparisons test (**c**, **d**). Areas highlighted in white indicate the light phase (ZT0-12) and areas highlighted in gray indicate the dark phase (ZT12-24). WT and APP-control animals were treated with the N-terminal PEmax vector encoding the *Dnmt1* epegRNA expression cassette. APP-treated animals were injected with PE3bmax/epegRNA1 targeting *Adrb1*. Each data point represents one animal (**b**, **d**, **e**). ZT, zeitgeber; min, minute; h, hour; REM, rapid eye movement; NREM, non-rapid eye movement; Hz, Hertz; EEG, electroencephalography; EMG, electromyography. Source data are provided as a Source Data file.

We delivered AAV-PHP.eB particles for installation of the *Adrb1*^A187V^ mutation (AAV-PE3bmax-nT and AAV-synth-W3-cT; see Fig. 2a for vector details) to the lateral ventricles of newborn APP-PS1 mice (Fig. 5a; hereafter referred to as APP-treated), which exhibit early onset (6 weeks of age) and fast progression of amyloid plaque deposition and gliosis^57^. At 15 weeks post-injection, we continuously recorded EEG/EMG signals (Supplementary fig. 20a) for three consecutive days where the animals were undisturbed with *ad libitum* food and water under a regular 12:12 light/dark cycle. Wild-type and transgenic APP-PS1 littermates, treated with the N-terminal PEmax vector encoding the *Dnmt1* pegRNA cassette (AAV-PEmax-nT; see Fig. 2a for vector details), were used as healthy (hereafter referred to as WT) or diseased controls (hereafter referred to as APP-control), respectively.

Analysis of EEG signals revealed a robust distribution of sleep/wake patterns across the day, with on average 66.9% (WT), 69.5% (APP-control), or 65.6% (APP-treated) of sleep occuring during the light phase (Fig. 5b; supplementary fig. 20b). Compared to WT, total sleep amounts were not significantly changed in APP-control animals during the light (WT, 427.0 ± 6.5 min; APP-control, 429.0 ± 12.6 min; Fig. 5b) and dark phase (WT, 215.0 ± 14.7 min; APP-control, 188.3 ± 9.0 min; Fig. 5b). Nevertheless, APP-control animals displayed a significant increase in REM sleep during the light phase (WT, 68.3 ± 3.2 min; APP-control, 77.4 ± 0.9 min), similar to recent observations in APPswe/PSEN1dE9 mice^58^, and a trend towards increased wakefulness (WT, 506.3 ± 14.7 min; APP-control, 523.4 ± 15.6 min; Fig. 5b) and less NREM sleep (WT, 199.5 ± 12.5 min; APP-control, 172.7 ± 8.7 min; Fig. 5b) during the dark phase, which aligns with previous reports of several AD models with APP overexpression^59^. Upon introduction of the *Adrb1*^A187V^ mutation, we detected a restoration of physiological levels of REM sleep during the light phase (WT, 68.3 ± 3.2 min; APP-control, 77.4 ± 0.9 min; APP-treated, 61.0 ± 4.5 min; Fig. 5b) and a trend towards physiological amounts of wakefulness (WT, 506.3 ± 14.7 min; APP-control, 523.4 ± 15.6 min; APP-treated, 504.3 ± 16.9 min; Fig. 5b) and NREM sleep during the dark phase (WT, 199.5 ± 12.5 min; APP-control, 172.7 ± 8.7 min; APP-treated, 197.1 ± 16.1 min; Fig. 5b), with higher prime editing efficiencies resulting in more pronounced changes in wakefulness (r = 0.9367, *p* = 0.0059) and NREM sleep (r = −0.9443, *p* = 0.0046; supplementary fig. 21). Similar to our observations in healthy mice (Fig. 3d,e; supplementary fig. 10), installing the *Adrb1*^A187V^ mutation in APP-PS1 mice did not alter REM spectra (Supplementary fig. 20c), but led to a consistent reduction of NREM sleep in the delta frequency range (between 2.5–3.5 Hz; Fig. 5c). Again, the reduction in NREM sleep EEG delta power was most pronounced in the dark phase (Fig. 5c; supplementary fig. 20d) and became more apparent with increasing prime editing efficiencies (r = 0.8302, *p* = 0.0408; Fig. 5d; supplementary fig. 21). Off-note, compared to APP-control animals, we also detected a significant decrease in NREM sigma (σ; 10–15 Hz) power, which has previously been shown to negatively correlate with locus coeruleus (LC) activity^60,61^, potentially inferring higher LC activity in APP-treated mice (Supplementary fig. 22).

Finally, we assessed if installation of the *Adrb1*^A187V^ mutation in APP-PS1 mice also altered amyloid plaque deposition or microglia activation (Fig. 5a). After whole-brain tissue clearing and immunolabeling using a modified iDISCO (immunolabeling-enabled three-dimensional imaging of solvent-cleared organs) protocol^62,63^, we aligned the collected data to the Allen mouse brain reference atlas (mouse.brain-map.org; supplementary fig. 23a) and segmented β-amyloid plaques and plaques engulfed by microglia (Supplementary fig. 23b). However, three-dimensional histological analysis revealed no significant changes in plaque progression and microglia pathology across the examined brain regions at 17 weeks post-injection (Fig. 5e, f; supplementary fig. 23c). Thus, while introduction of the *Adrb1*^A187V^ mutation in newborn APP-PS1 mice restored physiological levels of REM sleep and reduced SWA in NREM sleep, it did not substantially alter other AD-associated pathologies.

## Discussion

While several studies have utilized Cas9 nucleases to disrupt gene functions in the brain^64–67^, programmable nucleases cannot be employed for installing precise modifications in the CNS due to the absence of HDR in non-dividing cells^68^. Base editors offer an alternative by facilitating direct base deamination without requiring HDR. Despite successful reports of base editing in non-dividing cells^35,69–76^, including neurons, this technology is limited to the installation of transition point mutations^77–79^. Furthermore, the presence of other targetable bases within the editing window frequently results in the introduction of unwanted bystander edits, which can potentially hamper therapeutic efficacy^80^. Similar to base editing, prime editing works independently of HDR, but additionally offers the advantage of installing any small-sized edit type without introducing unwanted bystanders^1^.

In our study, we developed an in vivo prime editing approach for the brain. By optimizing pegRNA designs and AAV vectors encoding for intein-split PEs, we achieved editing rates of up to 61.2% at the *Dnmt1* locus (10 weeks post-injection) and 28.1% (6 months post-injection) at the *Adrb1* locus in the cortex. Introducing the *Adrb1*^A187V^ mutation was greatly enhanced by using the PE3b approach, in which a second ngRNA is employed to cut the unedited strand after successful editing at the PAM strand. In addition, we observed that prime editing precision was drastically increased when the PAM was co-edited, likely because these edits circumvent re-targeting of the edited locus by the PE complex.

Introduction of the autosomal dominant short sleep *Adrb1*^A187V^ mutation into the brains of newborn C57BL/6 J mice resulted in significant changes in locomotion, exploration, recognition memory, neuronal excitability within the CA1 area of the hippocampus, and sleep quality as indicated by changes in SWA during NREM sleep. Moreover, installing the *Adrb1*^A187V^ variant into the brains of newborn APP-PS1 mice restored physiological REM sleep levels and reduced NREM sleep EEG delta power and SWA following prolonged spontaneous wakefulness. However, other AD-associated pathologies, including β-amyloid plaques and plaque-associated microglia, were not improved. As the severity of sleep impairments and associated neuropathologies vary highly across AD models and may undergo dynamic changes throughout disease progression^59^, it would be prudent to conduct comparable assessments of these neuropathologies in mouse models that better reflect human disease progression such as *App*^*NF-L*^ knock-in mice^81^. Such studies could help determine the potential therapeutic benefit of the *Adrb1*^A187V^ variant.

When we specifically introduced the *Adrb1*^A187V^ mutation into the mPFC or dHC of adult mice, we did not observe any behavioral phenotypes. Since Adrb1 exerts functions in various brain regions, such as the dorsal pons^23^ or central amygdala^24^, it is possible that interactions between *Adrb1*^A187V^ neuronal populations across multiple brain areas may be required to trigger these phenotypic changes. Nevertheless, given the consistent decrease in NREM sleep EEG spectral power, specifically at the delta band range, across different genetic backgrounds and experiments, our data clearly suggest that *Adrb1*^A187V^ neurons play an important role in modulating the build-up of NREM sleep SWA following periods of prolonged spontaneous wakefulness. In fact, previous functional analyses of WT and mutant *Adrb1*^A187V^ neurons using fiber photometry, EEG/EMG recordings, and electrophysiology revealed an increase in the population activity of *Adrb1*^A187V^ neurons in the dorsal pons during the active phase as well as increased excitability and excitatory glutamatergic neurotransmission^23^. As our field recordings in hippocampus slices confirmed the increased excitability of prime-edited neurons, it seems plausible that the reported changes in electrophysiological properties of *Adrb1*^A187V^ neurons may contribute to the various behavioral changes observed in this study.

While our study demonstrates that the *Adrb1*^A187V^ variant has beneficial effects on various behaviors under physiological and pathological conditions, further research is required to i) uncover the underlying molecular and cellular mechanisms, ii) assess the contributions of *Adrb1*^A187V^ neuronal populations in different brain regions, iii) evaluate changes in noradrenaline dynamics and LC activity in detail, and iv) explore the therapeutic potential of installing *Adrb1*^A187V^ or other FNSS mutations in a more representative AD mouse model at different stages of neurodegeneration. Additionally, it would be interesting to assess if pharmacological modulation of noradrenergic signaling could also restore physiological sleep in AD mouse models. Overall, our study demonstrates that prime editing can be a powerful tool for neuroscience and other disciplines to manipulate and understand genetic circuits in both physiological and pathological contexts.

## Methods

### Ethics statement

Animal experiments were performed following protocols approved by the Kantonales Veterinäramt Zürich and in compliance with the Swiss Animal Welfare Act and Protection ordinance.

### Generation of plasmids

To generate pegRNA plasmids, annealed spacer, scaffold, and 3’ extension oligos were cloned into the BsaI-digested pU6-pegRNA-GG- (Addgene #132777), pU6-tevopreQ1-GG- (Addgene #174038) or pU6-tmpknot-GG-acceptor plasmid (Addgene #174039) using Golden Gate assembly as previously described. ^1,10^. ngRNA plasmids were generated by ligating annealed and phosphorylated oligos into a BsmBI-digested lentiGuide-Puro (Addgene #52963) backbone using T4 DNA ligase (Addgene #8453). To generate intein-split PE plasmids, inserts were either ordered as gBlocks from Integrated DNA Technologies (IDT) or amplified from pCMV-PE2 (Addgene #132775) or pCMV-PEmax (Addgene #174820) plasmids using PCR. Inserts were cloned into the NotI- and EcoRI-digested pCMV-PE2 backbone using HiFi DNA Assembly Master Mix [New England Biolabs (NEB)]. To generate PiggyBac reporter plasmids for the *Adrb1* locus, inserts with homology overhangs for cloning were ordered from IDT and cloned into the XbaI- and EcoRI-digested pPB-Zeocin backbone using HiFi DNA Assembly Master Mix (NEB). To prepare plasmids for AAV production, inserts with homology overhangs were either ordered as gBlocks (IDT) or generated by PCR. Inserts were cloned into XbaI- and NotI-digested AAV backbones using HiFi DNA Assembly Master Mix (NEB). All PCRs were performed using Q5 High-Fidelity DNA Polymerase (NEB). The identity of all plasmids was confirmed by Sanger Sequencing. Primers used for cloning all plasmids are listed in supplementary Tables 1 and 2. Amino acid sequences of intein-split PEmax p.713/p.714 constructs are listed in supplementary Table 7.

### Cell culture transfection and genomic DNA preparation

HEK293T [American Type Culture Collection (ATCC) CRL-3216] and Hepa1-6 (ATCC CRL-1830) cells were maintained in Dulbecco’s modified Eagle’s medium (DMEM) plus GlutaMAX (Thermo Fisher Scientific), supplemented with 10% (v/v) fetal bovine serum (FBS) and 1% penicillin/streptomycin (Thermo Fisher Scientific) at 37 °C and 5% CO_2_. Neuro2a (ATCC CCL-131) cells were maintained in Eagle’s Minimum Essential Medium (EMEM), supplemented with 10% (v/v) FBS and 1% penicillin/streptomycin. Cells were passaged every 3 to 4 days and maintained at confluency below 90%.

Cells were seeded in 96-well cell culture plates (Greiner) and transfected at 70% confluency using 0.5 μl Lipofectamine^TM^ 2000 (Thermo Fisher Scientific). If not stated otherwise, 300 ng of PE, 100 ng of pegRNA, 40 ng of nicking sgRNA (where indicated), and 150 ng of dnMLH1 (Addgene #174824) were used for transfection. When intein-split PEs were transfected, 300 ng of each PE half was used. Cells were incubated for 3 days after transfection.

Genomic DNA from cells was isolated using a lysis buffer (10 mM Tris-HCl, 2% Triton™ X-100, 1 mM EDTA, proteinase K [20 mg/mL]; Thermo Fisher Scientific). Cells were lysed at 60 °C for 1 h, followed by 10 min at 95 °C. Lysates were further diluted with 60 µL of dH_2_O. Genomic DNA from mouse tissues was isolated by phenol/chloroform extraction. First, tissue samples were incubated overnight in lysis buffer (50 mM Tris-HCl pH 8.0, 100 mM EDTA, 100 mM sodium chloride, and 1% SDS; Thermo Fisher Scientific) at 55 °C and 300 rpm. Subsequently, phenol/chloroform/isoamyl alcohol (25:24:1, Thermo Fisher Scientific) was added and samples were centrifuged (5 min, maximum speed). The upper phase was transferred to a clean tube and DNA was precipitated using 100% ethanol (Sigma-Aldrich). Samples were centrifuged (5 min, maximum speed) and pellets were washed using cold 70% ethanol (−20 °C). Washed pellets were dried at 55 °C for 10 min and resuspended in 100 µL of dH2O.

### Generation of cell lines with the integrated Adrb1 target site

To generate Adrb1 cell lines with the PiggyBac transposon, HEK, Hepa, and N2a cells were seeded into a 48-well cell culture plate (Greiner) and transfected at 70% confluency with 225 ng of the PiggyBac-transposon and 25 ng of the transposase using Lipofectamine 2000 (Thermo Fisher Scientific) according to the manufacturer’s instructions. Three days after transfection, cells were enriched for 10 days using Zeocin selection [150 μg/ml].

### AAV production

For the production of a pseudo-typed vector (AAV2 serotype PHP.eB) expressing EGFP under the control of the Cbh promoter, 2 × 10^8^ HEK293T cells were seeded per 150 mm dish (5 dishes in total) 24 h prior to transfection. For transfection, the helper plasmid (25 μg per dish), capsid plasmid (15 μg per dish) and cargo plasmid (9 μg per dish) were mixed with serum-free DMEM (Thermo Fisher Scientific) and polyethylenimine (PEI, Polyscience) was added in a 1:3 ratio (1 mg of DNA to 3 mg of PEI). The mix was incubated for 20 min at RT and then added to the cells. After 5 days of incubation at 37 °C and 5% CO2, cells were harvested and centrifuged for 15 min at 1500 × *g* in a conical corning flask (Thermo Fisher Scientific). 150 mL of the supernatant were mixed with 22 mL of NaCl (5 M) and 30 mL of PEG8000 (VWR) in a new corning flask. The cell pellet was resuspended in 4 mL of resuspension buffer (150 mM NaCl, 50 mM Tris-HCl, pH 8.0) and homogenized using a Precellys Evolution homogenizer (2 cycles: 5000 rpm for 45 sec with 15 s break). 300 units of benzonase (Sigma-Aldrich) were added to the disrupted cells and the mixture was incubated for 30 min at 37 °C in a water bath. After centrifugation at 5000 × *g* for 1 h, the supernatant was collected and mixed with the supernatant after harvesting. AAV particles in this mixture were precipitated for 2 days at 4 °C, followed by centrifugation at 10’000×g for 30 min. The supernatant was discarded and the AAV particles were washed using 4 mL of resuspension buffer. Particles were resuspended in 1.5 mL of NaCl [5 M]. Next, four fractions of OptiPrep GradientDensity medium (Sigma-Aldrich) were prepared (15%, 25%, 40%, and 60%). The most concentrated fraction was prepared at the bottom of the ultracentrifugation tube and least concentrated fraction at the top of the tube. The virus suspension was added at the top of the tube and the gradient was ultracentrifuged at 65,000 rpm at 15 °C for 2 h. AAV particles were harvested from the 40% gradient fraction and filtered using a pre-washed 100 kDa Amicon (Vivaspin). Virus particles were subsequently washed multiple times with PBS (pH 7.4, Thermo Fisher Scientific) and physical titers were measured using a Qubit 3.0 fluorometer and the Qubit dsDNA HS assay kit (Thermo Fisher Scientific).

All other pseudo-typed vectors (AAV2 serotype PHP.eB) were produced by the Viral Vector Facility of the Neuroscience Center Zurich. Briefly, AAV vectors were ultracentrifuged and diafiltered. Physical titers (vector genomes per milliliter, vg/mL) were determined using a Qubit 3.0 fluorometer (Thermo Fisher Scientific) as previously published^82^. The identity of the packaged genomes of each AAV vector was confirmed by Sanger sequencing.

### Negative staining and electron microscopy

First, carbon-coated electron microscopy (EM) grids (200 mesh, Quantifoil) were glow-discharged. Grids were briefly washed with a drop of 0.01% bovine serum albumin (BSA, Sigma-Aldrich). Subsequently, 2 μL of the sample was applied to the grid and incubated for 5 min. Excess liquid was removed from the edge of the grid with filter paper (Whatman). Next, grids were washed with 1 mM EDTA (Sigma-Aldrich), followed by staining with 0.5% uranyl acetate for 1 min. The liquid was again removed from the edge of the grid with filter paper and grids were dried overnight before imaging. Data were collected using an FEI Talos 120 kV transmission electron microscope (Thermo Fisher Scientific) equipped with a digital CMOS camera. Micrographs of several grid squares were collected for each AAV preparation to determine the ratio of fully packaged, partially packaged, and empty AAV particles. Data were quantified using MAPS (Thermo Fisher Scientific) and Fiji (version 2.14.0)^83^.

### Animal studies and injections

C57BL/6 J (obtained from the laboratory animal service center [LASC] in Zurich), C57BL/6 JRj (obtained from the ETH Phenomics Center), and double transgenic APP-PS1 [B6.Cg-Tg(Thy1-APPSw, Thy1-PSEN1*L166P)21Jckr] mice (obtained from the LASC in Zurich) were housed in IVC cages (2–5 animals per cage) in a pathogen-free facility at the University of Zurich. All mice were kept in a temperature- ( ~ 20 °C) and humidity-controlled ( ~ 40-60%) room on a 12:12 light/dark cycle. If not stated otherwise, mice were kept in closed standard cages with metal grids. Mice were fed a standard laboratory chow (Kliba Nafag no. 3437 with 18.5% crude protein). Food and water were provided *ad libitum*. Group assignment was semi-randomized.

Unless stated otherwise, newborn C57BL/6J and APP-PS1 mice (P1) received 5.0 × 10^10^ vg per animal and construct via intracerebroventricular (ICV) injection. Adult male C57BL/6JRj mice at P60-P90 were used to perform surgeries for the delivery of PE-AAVs to specific brain regions (dose of up to 1.0 × 10^9^ vg per hemisphere). Buprenorphine [0.1 mg/kg body weight] or Meloxicam [5 mg/kg body weight] was administered to mice subcutaneously 30 min prior to surgery. A local anesthetic [Emla cream: 5% lidocaine, 5% prilocaine] was additionally applied before the surgery. Animals were anesthetized using isoflurane (2% isoflurane with 1000 mL/min in 100% O_2_) and placed into a stereotaxic mouse frame on a warming surface to maintain body temperature. Anesthesia was maintained at 1.5–2.5% isoflurane with 400 mL/min in 100% O_2_ during surgeries. The skull was exposed and bregma was located. For experiments with adult C57BL/6 J mice, AAVs were injected bilaterally into the medial prefrontal cortex (mPFC). A small hole was drilled at the following coordinates (relative to bregma): 1.8 mm anteroposterior (AP); ±0.4 mm mediolateral (ML); −1.8 mm dorsoventral (DV). 0.8–1 μL injections per hemisphere were performed at a speed of 50 nL/min using a pneumatic injector (Narishige) and calibrated microcapillaries (Sigma-Aldrich). The needle was slowly removed 5 min after injection and the wound was sutured using Vicryl 5-0 suture (Ethicon). Animals received additional doses of buprenorphine [0.1 mg/kg body weight] or meloxicam [5 mg/kg body weight] after the surgery and on the next morning. The health of all animals was evaluated by post-operative checks over the course of 3 consecutive days.

Behavior experiments and activity monitoring of C57BL/6 J mice were performed at 4-5 weeks post-injection. Newborn C57BL/6 J mice were euthanized at 5, 10, or 24 weeks of age. Adult C57BL/6JRj mice were euthanized at 16 weeks of age. Newborn APP-PS1 mice were euthanized at 17 weeks of age.

### Behavioral assays

Before performing behavior experiments, all animals were handled for three days to get used to the experimenter. For animals injected as newborns, all tests were conducted under background dim illumination (intensity 30 Lx) in the light phase, and under dim red light (intensity 30 Lx) in the dark phase on consecutive days. For animals injected as adults, all tests were conducted under dim red light in the dark phase on consecutive days. Mice remained group-housed through the experiments, if not stated otherwise. Tests were performed at least 2 h after the respective light phase onset and finished at least 2 h before the onset of the next light/dark cycle. To minimize the stress of mice, animals were brought into the experimental room in their home cages at least 1 h prior to the test.

### Open field test

For the open field (OF) test, experiments with mice injected as adults were performed as previously described.^45^. Briefly, OF tests were conducted in sound insulated, ventilated multi-conditioning chambers (MultiConditioning System, TSE Systems Ltd, Germany). The OF arena (45 × 45 × 40 cm) consisted of four transparent plexiglas walls and a light gray PVC floor. Mice were recorded for 10 min from the top under dim illiumination (intensity of 4 Lx across the floor of the OF, provided by four equally spaced overhead lights). After the experiment, mice were returned to their home cage. DeepLabCut 2.0.7 was used to track the performance of all animals in an automated manner.

For experiments with mice injected as newborns, a 50 × 50 × 50 cm chamber made of black plastic walls and a white floor was used. Mice were recorded for 10 min from the top (C270 HD Webcam, Logitech). Locomotor activity and time spent in the center was automatically quantified using a custom-written MATLAB script. The average speed of a mouse was calculated as the distance covered during the running time divided by the time the mouse spent running. Running ‘episodes’ were automatically detected as the time intervals when the instant velocity of the mouse was higher than a given threshold of 5 cm/s. Instant velocity of the mouse was calculated for a sliding window of 4 frames. Fecal boli and grooming events were quantified manually. After the experiment, mice were returned to their home cage.

### Cylinder test

For the assessment of explorative behavior, mice injected as newborns were placed in a plexiglas cylinder (10 cm diameter, 30 cm height) and recorded for 10 min from the side after a 2 min habituation. After the experiment, mice were returned to their home cage. Wall-supported rearings in the cylinder were manually quantified in the light phase.

### Light dark transition test

For mice injected as adults, experiments were performed in sound insulated, ventilated multi-conditioning chambers (MultiConditioning System, TSE Systems Ltd, Germany) as previously described^84^. Briefly, the light dark box (LDB) consists of a box with a light (28 × 30 × 25 cm, illumination intensity of 200 Lx) and a dark (16 × 30 × 25 cm, illumination intensity of 0-1 Lx) compartment. The compartments were separated by a black plexiglas wall with a small central opening (6.6 × 7 cm) to let the mouse move freely between the compartments. The entire arena had a light gray PVC floor. Animals were placed directly into the center of the light compartment and recorded for 10 min. After the experiment, mice were returned to their home cage. DeepLabCut 2.0.7 was used to track the performance of all animals in an automated manner.

For mice injected as newborns, mice were first placed in the dark compartment (20 × 20 × 15 cm), which was covered with an opaque lid and recorded for 10 min. The light box (20 × 20 × 15 cm) was covered with a transparent top. After the experiment, mice were returned to their home cage. Behaviors were quantified manually.

### Elevated plus maze

The elevated plus maze (EPM) test was performed in a plus maze, elevated to 57 cm above ground (open arms: 30×5.5×0.3 cm; closed arms: 30×5.5×15 cm). The maze was made of gray plastic and open arms were illuminated with dim white light at an illumination intensity of 20 Lx. Animals injected as adults were placed on the center platform and recorded for 5 min. After the experiment, mice were returned to their home cage. DeepLabCut 2.0.7 was used to track the performance of all animals in an automated manner.

### Marble-burying test

Mice injected as newborns were placed into a new home cage (40 × 28 × 15 cm), filled with 5 cm of wood chip bedding, and covered with a transparent lid. 20 glass marbles were evenly placed in the cage to ensure equal and consistent positioning. Mice were exposed to the marbles for 30 min while recorded from the top. After the experiment, mice were returned to their original home cage. Behaviors were quantified manually.

### Novel object recognition

For analysis of memory performance, the same OF arena as described in section “Open field test” for newborns was used. On day 0, animals were first habituated to the OF arena for 10 min. On day 1, animals were placed into the arena and were exposed to two identical objects for 10 min. On day 2, one of the objects was replaced and animal behavior was recorded from the top for 10 min. After the experiment, mice were returned to their original home cage. Behaviors were quantified manually.

### Infrared activity in the home cage

For monitoring the home cage activity of mice using infrared (IR) sensors, animals were single-housed and an IR sensor was placed on top of the home cage. After 1-week of habituation, activity was recorded for at least 5 consecutive days and data were analyzed using ClockLab (Actimetrics).

### Behavioral data analysis using Deep Lab Cut

For all behavioural tests, DeepLabCut 2.0.7 was used to track the performance of the animals. The data generated by DeepLabCut was processed using custom R Scripts that are available online (https://github.com/ETHZ-INS/DLCAnalyzer). More detailed information is available in the original publication^85^.

### EEG/EMG implantation and recordings

Due to the legal constraints of single-housing female mice, male mice were exclusively used for EEG/EMG experiments. Mice were anesthetized using isoflurane as described in section “Animal studies and injections”. For EEG recordings of C57BL/6 J and APP-PS1 mice injected as newborns, 5 gold-plated intracranial EEG screws (0.9 mm diameter) were implanted on the mouse skull; 3 screws were used for the recordings and 2 screws for fixation of the implant. EEG screws were implanted at the following coordinates relative to bregma: 1.5 mm (ML) and 1.2 mm (AP) for the right frontal lobe, −1.8 mm (ML) and −1.8 mm (AP) for the left parietal lobe, and above the cerebellum for the reference. For EMG recordings, two golden wires were inserted bilaterally into the mouse neck muscle. Electrodes and EMG wires were subsequently connected to stainless steel wires (0.07 mm thickness) and soldered to a fine cable. Last, the electrodes were fixed to the mouse skull using dental cement. Mice were single-housed in open-top cages with higher walls to allow cable attachment to a 360° rotary custom-made swivel and free movement during the whole recovery and recording periods. Food and water were provided *ad libitum* inside these cages and all mice were kept in a 12:12 light/dark cycle. Mice were allowed a recovery/habituation period of at least 7 days before EEG/EMG signals were recorded. All recordings were started at light onset (ZT0) and lasted for 3 days (C57BL/6 J or APP-PS1 mice).

### EEG/EMG signal processing and data analysis

Signal acquisition parameters were set using the software SignalExpress NI 2015 controlled via LABVIEW. Signals were amplified using a factor of ~2000, analog band-pass filtered (high pass filter: –3 dB at 0.016 Hz; low pass filter: –3 dB at 128 Hz), sampled at 10240 Hz, then decimated and stored at 512 Hz resolution. EEG and EMG signals were digitally filtered (band-pass, 0.1-48 Hz for EEG; 10–30 Hz for EMG) using the MATLAB function “cheby2”, down-sampled, and stored at 128 Hz resolution. Next, EEG spectral power was calculated for 4s-epochs using p-welche estimate (0.25 Hz resolution bins with no overlap Hanning window). Adjacent 0.25 Hz bins were averaged and stored as 0.5 Hz bins (for frequencies 0.5-5 Hz) or 1 Hz bins (for frequencies between 5.25-25 Hz)^86,87^. The different vigilance states (NREM sleep, REM sleep, and wakefulness) were automatically scored using the automated scoring platform SPINDLE, a previously validated machine learning-based system^88^, followed by visual inspection of the raw traces to ensure accuracy. NREM sleep was identified by the presence of high-amplitude slow waves and delta activity, while REM sleep was characterized by rhythmic theta activity. Epochs showing mixed slow wave and theta activity were classified as “transitions to REM” and were included in the quantification of total REM sleep durations (Supplementary Fig. 24). Representative examples for raw EEG/EMG traces in different vigilance states are shown in supplementary Fig. 9, 17, and 24. For the spectral power analysis during the different vigilance states, each frequency was normalized to the summed power of that frequency in the NREM sleep, REM sleep, and wakefulness in the 12 h light phase of recording day 1 as previously published^89^. This analysis strategy accounts for interindividual differences in both absolute spectra and in time spent in the different vigilance states while also allowing for additional observations between state-specific spectra. For comparison, we also analyzed power spectra of each vigilance state by normalizing to the total power of the same vigilance state, yielding similar albeit less pronounced changes (Supplementary Fig. 25). For the SWA timecourse analysis, delta power was normalized to the NREM EEG SW power during the last four hours of the light period (ZT8-12) from recording day 1. “Transitions to REM” were excluded from spectral anaylsis of both NREM and REM sleep. All analyses were performed in MATLAB (MathWorks R2020b) using custom-made scripts.

### Trans-cardiac perfusion, brain isolation, and dissection of brain regions

Sodium pentobarbital (Kantonsapotheke Zürich) was injected via intraperitoneal injection at a dose of 100 mg/kg. Complete anesthesia was confirmed by the absence of a toe pinch reflex. Mice were placed on the perfusion stage inside a collection pan and the peritoneal cavity was exposed. The diaphragm was cut through laterally and the rib cage was cut parallel to the lungs, creating a chest “flap”. The flap was clamped in place using a hemostat (Fine Science Tools) and a 25 gauge needle (Sterican), attached to silicon tubing and a peristaltic pump, was inserted into the left ventricle. The right atrium was cut for drainage. Animals were first perfused with ice-cold PBS (Thermo Fisher Scientific) at a rate of 10 mL/min, followed by perfusion with ice-cold fixative (4% paraformaldehyde, PFA, Sigma-Aldrich). When brains were used for a single cell, DNA, RNA, or protein isolation, perfusion was performed exclusively with PBS. Once the perfusion was complete, mice were decapitated and the skull was removed with scissors and tweezers without inflicting damage to the underlying tissue. The brain was removed using a spatula.

For the dissection of brain regions, PBS-perfused brains were first rinsed in PBS and then cut into 1 mm slices using an acrylic mouse brain matrix (AgnThos) and razor blades. The olfactory bulb, cortex, hippocampus, striatum, thalamus, hypothalamus, midbrain, hindbrain, and cerebellum were identified based on the mouse brain atlas^90^. To dissect *Adrb1*-expressing regions with high precision, 60 μm sections of PBS-perfused brains were prepared, and the region of interest was isolated under a stereomicroscope using the mouse brain atlas^91^.

For immunohistochemistry of brain sections, PFA-perfused brains were post-fixated in 4% PFA for 4 h (C57BL/6 J) or 16 h (APP-PS1), followed by overnight incubation in 30% sucrose.

### RNA isolation and RT-qPCR

RNA was isolated from cultured or isolated cells or snap-frozen brain tissues using the RNeasy Mini Kit (Qiagen) or the RNeasy Lipid Tissue Mini Kit (Qiagen) according to the manufacturer’s instructions. RNA (1000 ng input) was subsequently reverse-transcribed to cDNA using random primers and the GoScript RT kit (Promega). RT-qPCR was performed using FIREPoly qPCR Master Mix (Solis BioDyne) and analyzed using a Lightcycler 480 system (Roche). Fold changes were calculated using the ΔCt method. Primers used for RT-qPCR are listed in supplementary Table 3.

### Single-cell isolation by MACS

Cortices from PBS-perfused brains were cut into small pieces and dissociated using the Adult Brain Dissociation Kit and gentleMACS Octo Dissociator with heaters (Miltenyi Biotec) according to the manufacturer’s instructions. Cell debris and myelin was subsequently removed using a debris removal solution (Miltenyi Biotec). The single-cell suspension was used for the isolation of neurons using the Adult Neuronal Isolation kit (Miltenyi Biotec) according to the manufacturer’s instructions. The identity of all fractions was confirmed by flow cytometry and RT-qPCR. Briefly, cells were resuspended in FACS buffer (PBS supplemented with 2% FBS and 2 mM EDTA). Fc blocking reagent (1:50 in FACS buffer) was added to all samples and incubated on ice for 20 min. Samples were subsequently labeled with primary antibodies (ACSA-2, O4, CD11b, Biotin) and a viability dye (eFluor780), diluted in FACS buffer, for 1 h at 4 °C (in the dark). Cell suspensions were washed three times in FACS buffer and filtered through a 35 μm nylon mesh cell strainer snap caps (Corning) and kept on ice until analysis. For each sample, 10,000-50,000 events were counted on an LSRFortessa (BD Biosciences) using the FACSDiva software version 8.0.1 (BD Biosciences). Experiments were performed with three replicates/mice. The gating strategy is shown in supplementary fig. 8. RT-qPCR primers and antibodies, used to validate the identity of all samples, are listed in supplementary Tables and 4.

### Amplification for deep sequencing

Genomic DNA from cultured cells or brain tissues was isolated by direct lysis (cells) or phenol/chloroform extraction (brain tissue). *Adrb1-* or *Dnmt1*-specific primers were used to generate targeted amplicons for deep sequencing. Input genomic DNA was first amplified in a 10 μL reaction for 30 cycles using NEBNext High-Fidelity 2×PCR Master Mix (NEB). Amplicons were purified using AMPure XP beads (Beckman Coulter) and subsequently amplified for eight cycles using primers with sequencing adapters. Approximately equal amounts of PCR products were pooled, gel purified, and quantified using a Qubit 3.0 fluorometer and the dsDNA HS Assay Kit (Thermo Fisher Scientific). Paired-end sequencing of purified libraries was performed on an Illumina Miseq. Primers for deep sequencing are listed in supplementary Table 5.

### HTS data analysis

Sequencing reads were first demultiplexed using the Miseq Reporter (Illumina). Next, amplicon sequences were aligned to their reference sequences using CRISPResso2^91^. Prime editing efficiencies were calculated as percentage of (number of reads containing only the desired edit)/(number of total aligned reads). Indel rates were calculated as percentage of (number of indel-containing reads)/(total aligned reads). Reference sequences are listed in supplementary Table 6.

### Electrophysiology recordings in brain slices

Newborn mice of both genders were used for all recordings. At 9–11 weeks post-injection, animals were decapitated under anesthesia, and the brains were quickly removed. Horizontal hippocampal mouse brain slices (300μm thick) were prepared in sucrose-based ACSF containing 85 mM NaCl, 1.25 mM NaH_2_PO_4_, 2.5 mM KCl, 24 mM NaHCO_3_, 4 mM MgCl_2_, 0.5 mM CaCl_2_, 75 mM sucrose, and 25 mM glucose. After incubation for 30 minutes at 35 °C, slices were transferred to physiological ACSF solution (126 mM NaCl, 1.25 mM NaH_2_PO_4_, 2.5 mM KCl, 2 mM MgCl_2_, 2 mM CaCl_2_, 26 mM NaHCO_3_, 10 mM glucose, saturated with 95% O_2_ and 5% CO_2_ at pH 7.4) and kept at room temperature. Field potential recordings were performed with low-resistance patch-clamp electrodes filled with ACSF. Field EPSPs were recorded in the stratum radiatum of area CA1. Schaffer collaterals were stimulated with a frequency of 0.05 Hz. Recordings were done in the presence of the β1-adrenoreceptor agonist dobutamine (10 μM) and, to attenuate field EPSP amplitudes, in the presence of sub-saturating concentrations of the AMPA/kainate receptor antagonist NBQX (100 nM).

### Immunohistochemistry

PFA-fixed brain tissues of C57BL/6 J mice were frozen on dry ice and cut into 40 μm-thick sections using a microtome. Sections were blocked in PBS supplemented with 2% normal donkey serum (cat. no. ab7475, abcam) and 0.3% Triton X-100 (Sigma-Aldrich) for 1 h. Brain sections were incubated with primary antibodies overnight at 4 °C (mouse-NeuN, 1:500, abcam ab177487; rabbit-Cas9, 1:1,000, Cell Signaling clone D8Y4K; chicken-GFAP, 1:1’500, abcam ab95231). Donkey anti-mouse-568 (1:500), donkey anti-chicken-647 (1:500) and donkey anti-rabbit-488 (1:1,000; all from Jackson ImmunoResearch) were used as secondary antibodies and sections were counterstained with 4′,6-diamidino-2-phenylindole (DAPI, Sigma-Aldrich). Mounting was performed using Prolong Gold Antifade Mountant (Thermo Fisher Scientific). Confocal images were taken with a Zeiss LSM 800 or a Zeiss AxioScan.Z1 slidescanner and analyzed with Fiji (version 2.14.0)^83^. Antibodies are listed in supplementary table 4.

### Tissue clearing and staining of mouse hemispheres

Mouse hemispheres were stained for microglia and amyloid beta plaques and cleared using a modified version of the iDISCO protocol^63^. Samples were dehydrated by serial incubations in 20%, 40%, 60%, and 80% methanol (MeOH) in ddH_2_O followed by two incubations in 100% MeOH, each for 1 h at RT and 40 rpm. Pre-clearing was performed in 33% MeOH in dichloromethane (DCM) overnight (o.n.) at RT and 40 rpm. After two 1h-washes in 100% MeOH at RT and 4 °C (40 rpm), bleaching was performed using 5% hydrogen peroxide in MeOH for 20 h at 4 °C (40 rpm). Samples were rehydrated by serial incubations in 80%, 60%, 40%, and 20% MeOH in ddH_2_O followed by incubation in PBS, each for 1 h at RT and 40 rpm. Permeabilization was performed by incubating mouse hemispheres two times in 0.2% TritonX-100 (Sigma-Aldrich) in PBS for 1 h (RT, 40 rpm), followed by incubation in 0.2% TritonX-100 supplemented with 10% dimethyl sulfoxide (DMSO; Sigma-Aldrich), 2.3% glycine (Sigma-Aldrich), and 0.1% sodium azide (NaN3; Sigma-Aldrich) in PBS for 3 days at 37 °C and 65 rpm. Blocking was subsequently performed in 0.2% Tween-20 (Sigma-Aldrich) supplemented with 0.1% heparine (10 mg/ml; Sigma-Aldrich), 5% DMSO, and 6% donkey serum in PBS for 2 days at 37 °C and 65 rpm. Next, samples were gradually stained with a primary polyclonal rabbit-anti-Iba1 antibody (1:400; Wako 019-19741), a secondary polyclonal 647-conjugated donkey-anti-rabbit antibody (1:400; ThermoFisher A-31573) and LCP cocktail hFTAA and qFTAA (1:1000) in a total of 1.5 mL staining buffer (0.2% Tween-20 supplemented with 0.1% heparine, 5% DMSO, and 0.1% NaN3 in PBS) per sample for 2 weeks at 37 °C and 65 rpm. Five 1h-washes with staining buffer were performed between and after the staining (RT, 40 rpm). A final wash performed for 1-2 days at RT and 40 rpm. Clearing was started by dehydrating the samples in serial MeOH incubations as previously described. Delipidation was performed o.n. in 33% MeOH in DCM at RT and 40 rpm followed by two incubation in 100% DCM, each for 20 min at RT and 40 rpm. Refractive index (RI) matching was achieved in dibenzyl ether (DBE, RI = 1.56) for 4 h at RT.

### Lightsheet imaging of cleared brain hemispheres

Whole-brain images of cleared brain hemispheres were recorded with a custom-made selective plane illumination microscope v.5 (www.mesospim.org)^92^ at a 2x zoom, providing a field of view of 1.3 cm and an isotropic resolution of 3 μm/voxel. Transparent brain hemispheres were imaged at a voxel size of 3.26 × 3.26 × 3 μm^3^ (x×y×z) with four scanning tiles per channel. For LCP-stained plaques, a 488 nm laser was used for excitation with a 520/35 nm bandpass filter. For Iba1-stained microglia, a 647 nm laser paired with a quadruple bandpass filter (405-488-561-640 nm). Both right and left lasers were used for data acquisition to reduce imaging artifacts.

### Image analysis of cleared brain hemispheres

Acquired tiles were stitched using the Big Stitcher plugin^93^ (spimdata version 0.9-revision; multiview-reconstruction version 3.2.5) in Fiji^83^ (version 2.14.0). Images were first converted from the.raw format to.nrrd to facilitate loading and processing with ImageJ. Pixel sizes were then verified to correspond to the acquisition settings of 3.26μm for the pixel width and height, and 3.0 μm between consecutively acquired z-planes. Next, 3D brains were spatially cropped to reduce the file size and computational time, and improve the alignment to the Allen Brain Atlas^94^. In particular, both channels of each sample (488 nm and 647 nm) were cropped using a rectangular prism across x,y,z coordinates to contain as little empty space around the sample as possible. The cropped data were then flipped accordingly such that the sagittal view of the data shows the outer edge of the brain hemisphere appearing at the beginning of the image stack, and the cerebellum on the left. In total, 15 samples were prepared out of which 4 were excluded from the analysis due to a lack of plaques or the presence of a hydrocephalus or staining artifacts.

Two pixel classifiers were trained using ilastik^95^. One was trained to detect plaques in the 488 nm channel while the other was trained to detect microglia in the 647 nm channel. The classifiers were both trained using cropped 3D chunks of both treatment groups, taken at different brain regions and depths to represent the spatial and cohort heterogeneity of plaques and microglia. Pixel classification was then performed on all samples, resulting in two probability maps per sample. One depicted the probability of each pixel to belong to a plaque and the other one depicted the probability of each pixel to belong to a microglia cell. Probability maps were then converted to a binary mask by setting all pixels with a probability higher than 0.2 to 1. A connected components algorithm with a 26-connectivity kernel was then applied to the binary masks using the MorphoLibJ’s plugin of ImageJ^96^, resulting in a list of plaque/microglia candidates. Morphological properties of the connected components were quantified using MorphoLibJ’s s “Analyze Regions 3D” functionality and stored in csv files for further processing. Additionally, the total, mean and standard deviation of the probability within each candidate were computed and appended to the csv data. Detected false plaque candidates were excluded by setting qualitatively selected thresholds to the minimum (2 voxels) and maximum plaque size (10000 voxels) as well as to the minimum ratio of the standard deviation over the mean probability per plaque candidate (0.1). Plaques engulfed by microglia were detected by performing a spatial colocalization of the true plaques with the binarized microglia.

Next, the 488 nm channel was used to align the data to the Allen brain reference atlas^94^ using the software elastix^97^. Prior to alignment, the data of the 488 nm channel were downsampled by a factor of 2 across all dimensions using an averaging operation. Then, the maximum intensity was capped at a manually selected threshold for each brain, which was qualitatively selected to reduce intensity outlier artifacts. Pixel noise was reduced by applying a 3D Gaussian blur with sigma 2 pixels per direction. These alignment-related preprocessing steps were found to improve alignment of the data to the Allen mouse brain reference atlas^94^. For alignment, the 25 mm voxel Allen brain reference mouse atlas was used. Due to difficulties in preserving an intact olfactory bulb during sample handling, the olfactory bulb was cropped out from the reference atlas. To register and align the data, a sequence of transformations was used. For the initial data registration, an affine transformation was applied followed by a b-spline transformation for aligning the reference atlas onto the sample data. A file containing all parameters for the elastix setup will be provided on Github upon publication. The centroids of all detected plaques were subsequently aligned using the same transformation approach.

### Quantification of plaques and plaque-associated microglia in cleared brain hemispheres

Total plaque counts were quantified in the following regions of the Allen brain anatomical atlas^94^: hippocampal formation, isocortex, thalamus, hypothalamus, midbrain, hindbrain. Medians and range of plaque counts were computed for each brain regions and treatment group. Additionally, the total volume of microglia which colocalized with detected plaques was quantified and reported as a fraction of the detected plaque volume. Last, density maps depicting the centroids and all detected voxels were plotted across selected coronal slices of the aligned data for plaques and microglia.

### Statistical analysis and reproducibility

All statistical analyses were performed using GraphPad Prism (version 10.2.1) for macOS. If not stated otherwise, data are represented as biological replicates and are depicted as means±standard deviation (s.d.). Statistical analyses are indicated in the corresponding figure legends. Likewise, sample sizes and the statistical tests performed are described in the respective figure legends. The data were tested for normality using the Shapiro-Wilk test if not stated otherwise. Unpaired two-tailed Student’s *t*-tests were performed followed by the appropriate post hoc test when more than two groups were compared. For all analyses, *P* < 0.05 was considered statistically significant. To determine sample sizes for animal experiments, a priori power calculations were performed using the R ‘pwr’ package. Except for EEG/EMG experiments, which were exclusively performed with male mice, treatment groups were randomly assigned without gender bias. For newborns, all animals of a litter were treated with the same AAVs and used for subsequent experiments. No animals were excluded from the study. For analysis of editing efficiencies, researchers were not blinded to group allocation. Blinding was not necessary here because the editing efficiency as a readout cannot be influenced by a biased researcher. All deep amplicon sequencing data was analyzed via an unblinded operator by using an automated script (CRISPResso2) with limited experimenter intervention. For performance and analysis of behavior and sleep experiments, researchers were blinded throughout the study. Biological replicates, animal numbers, and group sizes are indicated for each experiment in the respective figure legends. A detailed source data file containing the primary data and animal sexes is provided.

### Reporting summary

Further information on research design is available in the Nature Portfolio Reporting Summary linked to this article.

## Supplementary information

Supplementary Information

Reporting Summary

Transparent Peer Review file

## Source data

Source data

## Data Availability

Source data are provided with this paper. Illumina sequencing data generated in this study have been deposited in the Sequence Read Archive (SRA) database under BioProject number PRJNA1168859. The raw EEG/EMG data have been deposited on Zenodo (https://zenodo.org/records/16873341).  Source data are provided with this paper.
