# Supplementary material for: Prime editing of the β1 adrenoceptor in the brain restores physiological REM sleep in a mouse model of Alzheimer’s disease
Source: Nat Commun. 2025 Dec 9;16:10973. doi: 10.1038/s41467-025-65964-w (PMC12689628; doi:10.1038/s41467-025-65964-w)
Supplement: Supplementary file 1 — Supplementary Information [file 41467_2025_65964_MOESM1_ESM.pdf]

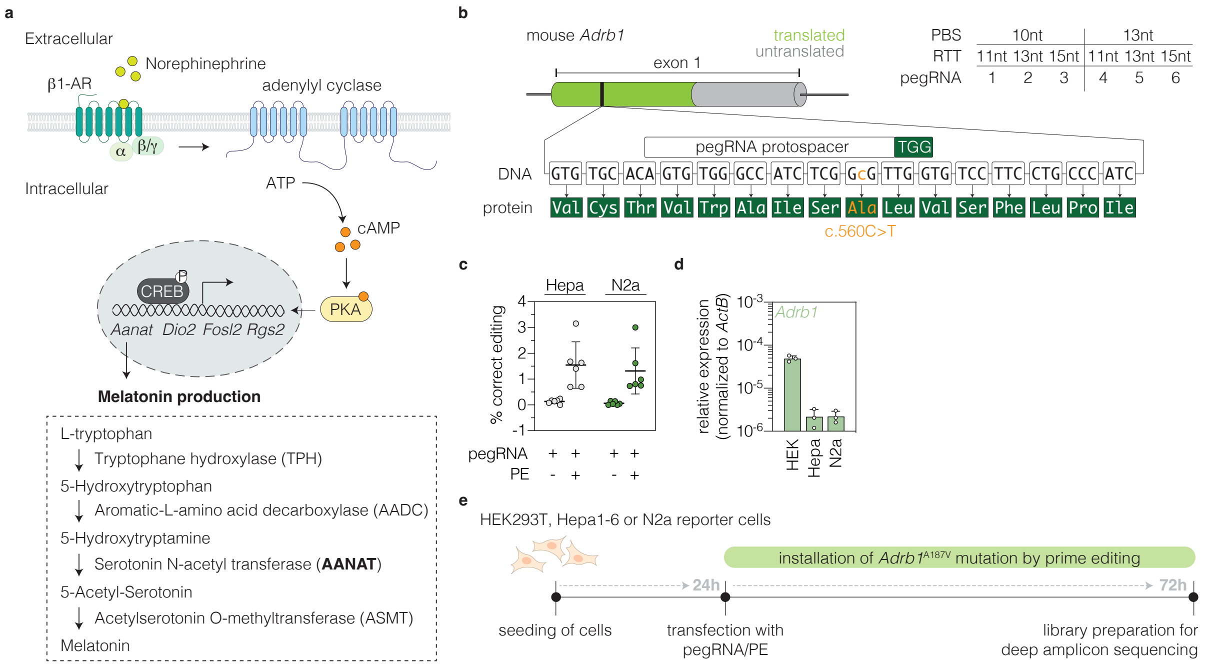

Supplementary figure 1 | Establishment of cell lines with the integrated *Adrb1* target site. (**a**) Schematic representation of β1-AR-mediated signaling and its potential link to wakefulness and sleep (created based on[83]). (**b**) Depiction of the *Adrb1* gene and pegRNA designs used at the targeted site (c.560C>T; p.A187V). (**c**) Prime editing rates of pegRNA1 at the endogenous *Adrb1* locus in murine Hepa and N2a cell lines (n=6 biological replicates per group). (**d**) *Adrb1* transcript levels in HEK, Hepa, and N2a cells (n=3 cell passages per group). Transcripts were normalized to *Gapdh*. (**e**) Schematic representation of the experimental timeline of *in vitro* prime editing experiments performed in this study. Data are represented as means±s.d. of independent experiments. β1-AR, β_1 adrenoceptor; ATP, adenosine triphosphate; cAMP, cyclic adenosine monophosphate; PKA, protein kinase A; CREB, cAMP responsive element binding protein; LTD/RTD, left/right transposable domains; EF1α, elongation factor alpha; bGH, bovine growth hormone; SV40, Simian virus 40; HSV-tk, Herpes simplex virus thymidine kinase; bp, base pairs. Data are represented as means±s.d. of at least three independent experiments (c) or three passages of cells (d).

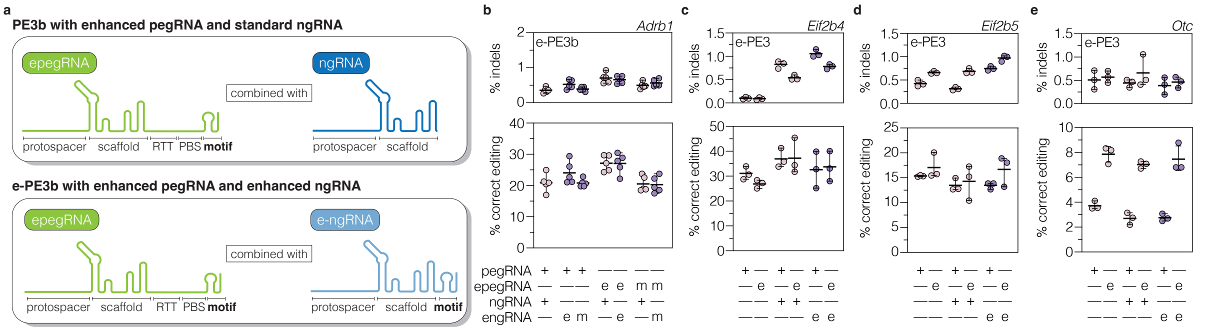

Supplementary figure 2 | Enhanced ngRNAs display similar *in vitro* performance to regular ngRNAs. (**a**) Schematic representation of the PE3/PE3b (epegRNA and ngRNA) and e-PE3/e-PE3b approach (epegRNA and engRNA). (**b-e**) e-PE3b (b) or e-PE3 (c-e) editing and indel rates at the *Adrb1* (b; n=5 biological replicates per group), *Eif2b4* (c; n=3 biological replicates per group), *Eif2b5* (d; n=3 biological replicates per group), and *Otc* (e; n=3 biological replicates per group) locus. Data are represented as means±s.d. and were analyzed using an unpaired two-tailed Student's t-test with Welch's correction. If not indicated, differences are not statistically significant (*P>0.05*). motif, stabilizing structural motifs; e, tevopreQ$_1$ motif; m, tmpknot motif.

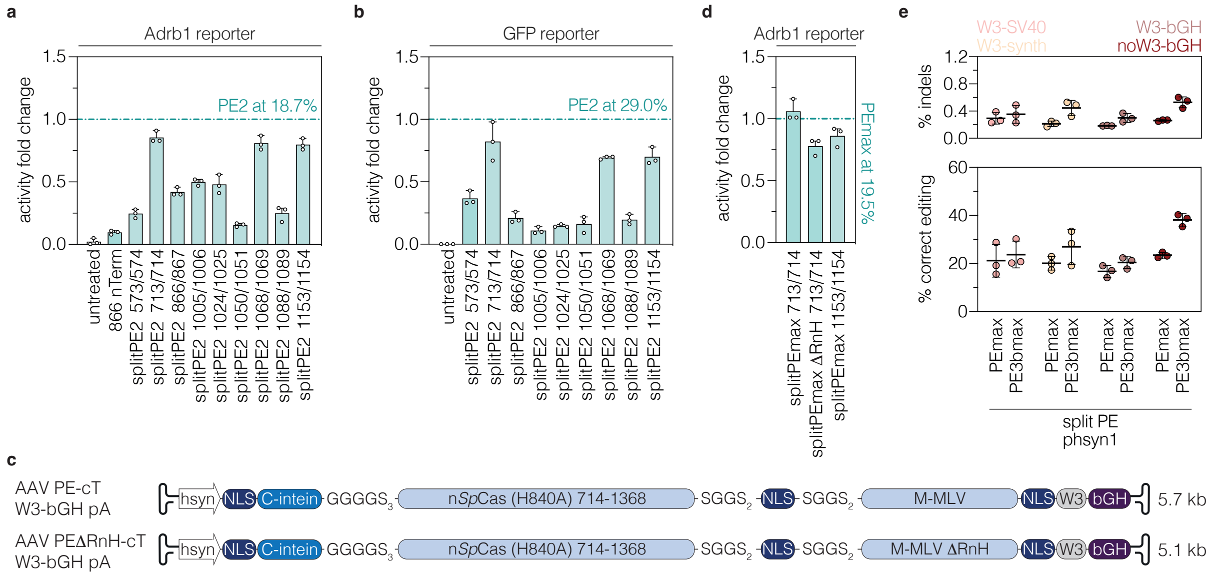

Supplementary figure 3 | *In vitro* refinement of intein-split PE variants and AAV vector designs for *in vivo* applications. (**a,b**) Relative performance of intein-split PE variants in HEK cells with the integrated *Adrb1* target site (a) or with an integrated GFP reporter locus (b). Performance was normalized to full-length PE2 (indicated as a dashed line). (**c**) Schematic representation of oversized c-terminal (cT) PE AAV vector constructs and the corresponding lengths in kilobase pairs (kb, including ITRs). Vectors are not depicted to scale. (**d**) Relative performance of intein-split PEmax variants in HEK cells with the integrated *Adrb1* target site. Performance was normalized to full-length PEmax (indicated as a dashed line). (**e**) Comparison of editing and indel rates of intein-split PEmax/PE3bmax AAV constructs under the control of the neuron-specific hSyn1 promotor. Data are represented as means±s.d. of 3 independent experiments. kb, kilobases; PEΔRnH, PE lacking RNaseH domain in RT; hSyn1, human synapsin 1 promoter; NLS, nuclear localization signal; W3, woodchuck hepatitis virus post-transcriptional regulatory element; SV40, Simian virus 40; synth, synthetic polyA, bGH, bovine growth hormone; RT, reverse transcriptase.

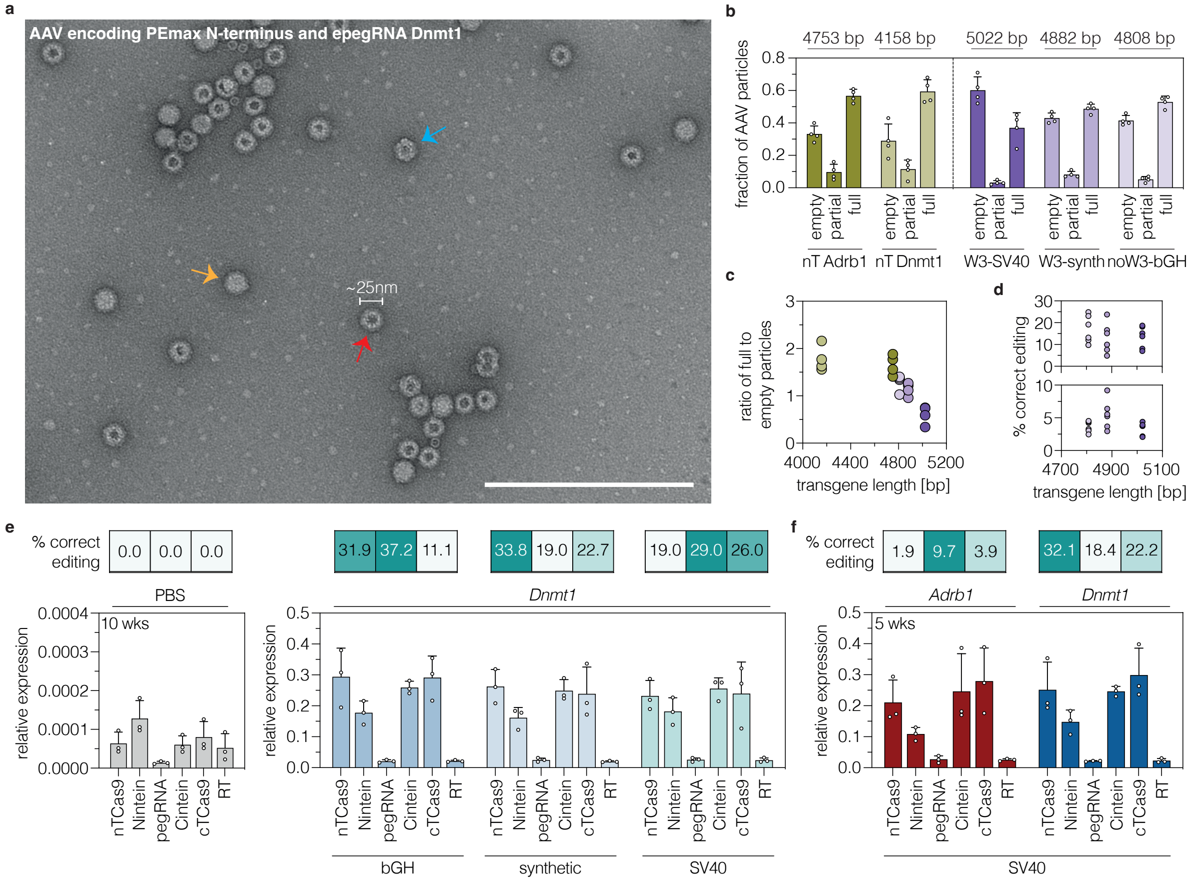

Supplementary figure 4 | Detailed *in vitro* and *in vivo* analyses of AAV preparations. (**a**) Representative electron micrograph (n=16) of negative-stained AAV particles (AAV-PEmax-nT with the epegRNA targeting *Dnmt1*) containing fully-packaged (orange arrow), partially-packaged (blue arrow), and empty particles (red arrow). Scale bar, 250 nm (n=4 individual grid squares). (**b-d**) Relationship between transgene length and packaging efficiency (b,c) or editing efficiency (d) at the *Dnmt1* and *Adrb1* locus. Transgene lengths in base pairs (bp) are indicated at the top above the respective construct (b). The color coding of AAV preparations in (c) and (d) corresponds to the same colors as in (b). (**e,f**) PEmax and epegRNA transcript levels of AAV-nT and -cT preparations targeting *Dnmt1* (e,f) or *Adrb1* (f) in mouse striata. Corresponding editing rates were determined by deep amplicon sequencing and are depicted for every animal above each AAV vector (e,f; n=3 mice per group). Transcript levels were normalized to *Gapdh*. Data are represented as means±s.d. (d-f). nT/cT, N-/C-terminal PEmax AAV vector; wks, weeks.

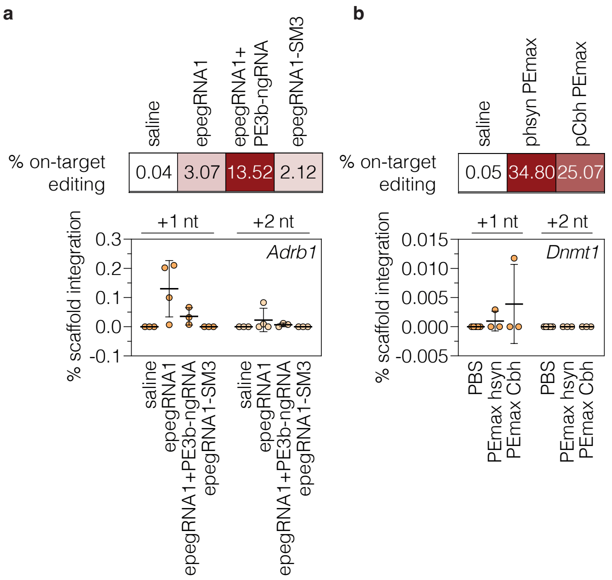

Supplementary figure 5 | Scaffold integrations are low at the *Adrb1* and *Dnmt1* locus. (**a,b**) Integration of the first (+1) and second (+2) nucleotide (nt) of the sgRNA scaffold into the genome at the *Adrb1* (a) and *Dnmt1* locus (b). Average on-target editing rates of the respective treatment groups are displayed on top. Data are represented as means±s.d. of 3 or 4 animals per group. Each data point represents one animal.

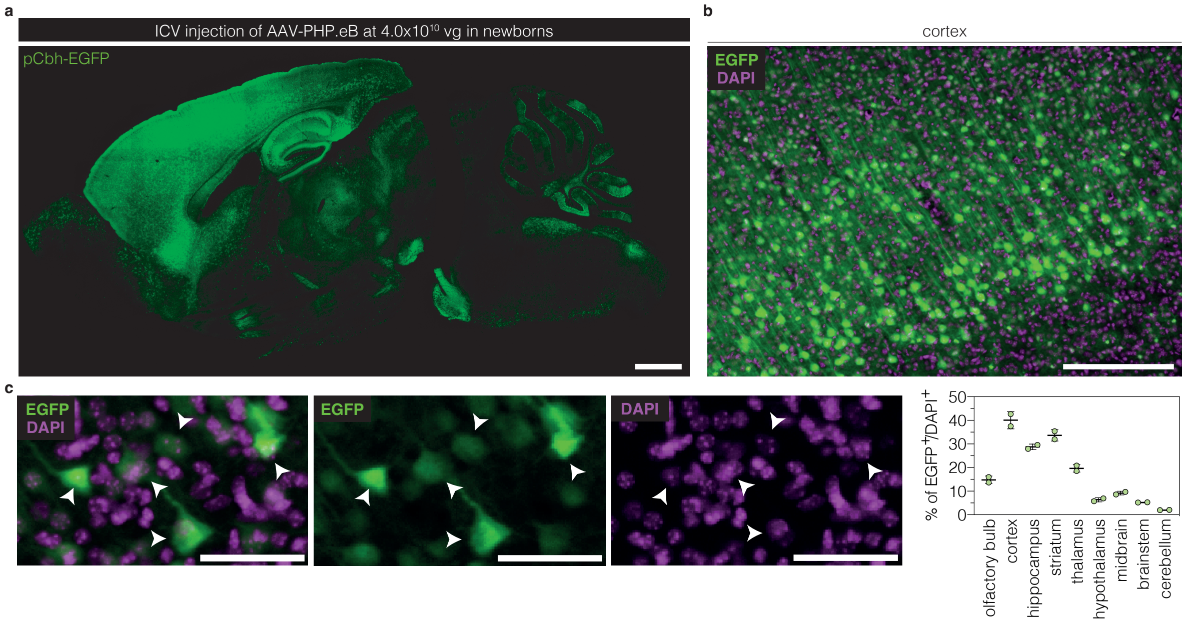

Supplementary figure 6 | Biodistribution of AAV-PHP.eB particles after ICV injection into newborn mice at postnatal day 1. (**a**) Representative whole-brain image (n=2 sections per animal) of EGFP fluorescence after 30 days of expression. AAV-PHP.eB particles, expressing EGFP under the Cbh promoter, were delivered to newborn mice at a dose of 4×10^{10} vg per animal via ICV injection. Image was taken from [73]. (**b,c**) Representative images of EGFP expression (green) and DAPI staining (magenta) in the cortex (b,c) and quantification of $DAPI^{+}$ cells (c) that were transduced in different brain regions. Data are displayed as means±range (n=2 mice). Scale bars, 1000 µm (a), 200 µm (b), and 50 µm (c). vg, vector genomes; EGFP, enhanced green fluorescent protein; DAPI, 4’,6-diamidino-2-phenylindole.

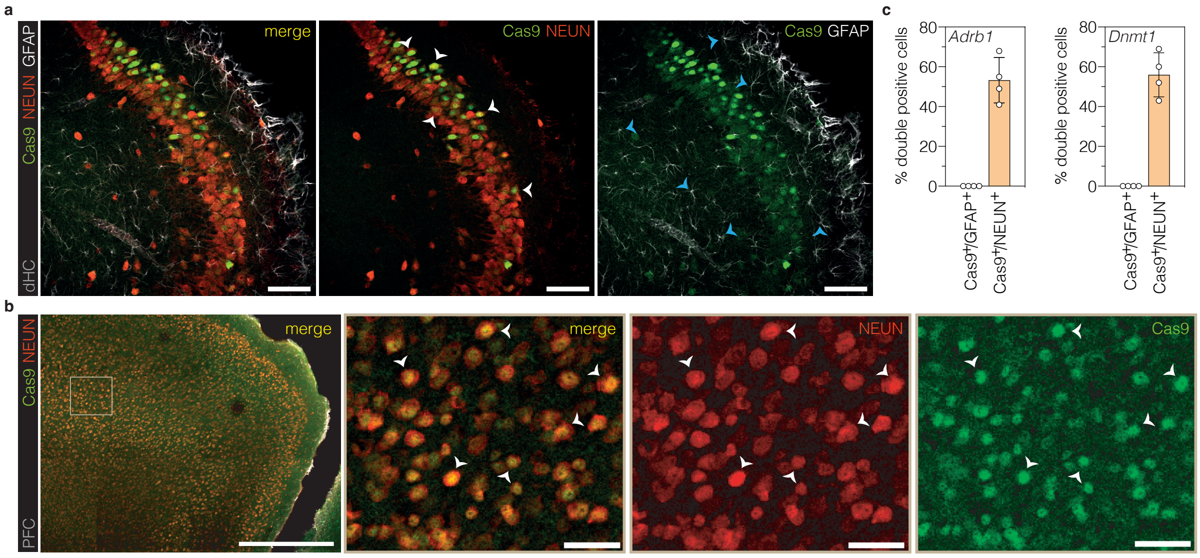

Supplementary figure 7 | Neuron-specific *Sp*Cas9 expression in the dorsal hippocampus and prefrontal cortex. (**a,b**) Representative fluorescence micrographs (n=2 sections per animal) of *Sp*Cas9, NEUN, and GFAP expression in the dorsal hippocampus (a, dHC) and *Sp*Cas9 and NEUN expression in the prefrontal cortex (b, PFC) of treated mice at 10 weeks post-ICV injection (n=1 mouse per locus). (**c**) Quantifications of the overlap between *Sp*Cas9 expression (Cas9, green) in neurons (NEUN, white arrowheads) and astrocytes (GFAP, blue arrowheads) in mice treated with hSyn1-PEmax targeting *Dnmt1* or hSyn1-PE3bmax targeting *Adrb1*. Data are represented as means±s.d. of 4 independently imaged tissue regions. At least 50 cells per cell type were used for quantification. dHC, dorsal hippocampus; PFC, prefrontal cortex; GFAP, glial fibrillary acidic protein; NEUN, hexaribonucleotide binding protein-3. Scale bars (left to right), 25µm (a), and 500µm, 20µm, 20µm, 20µm (b).

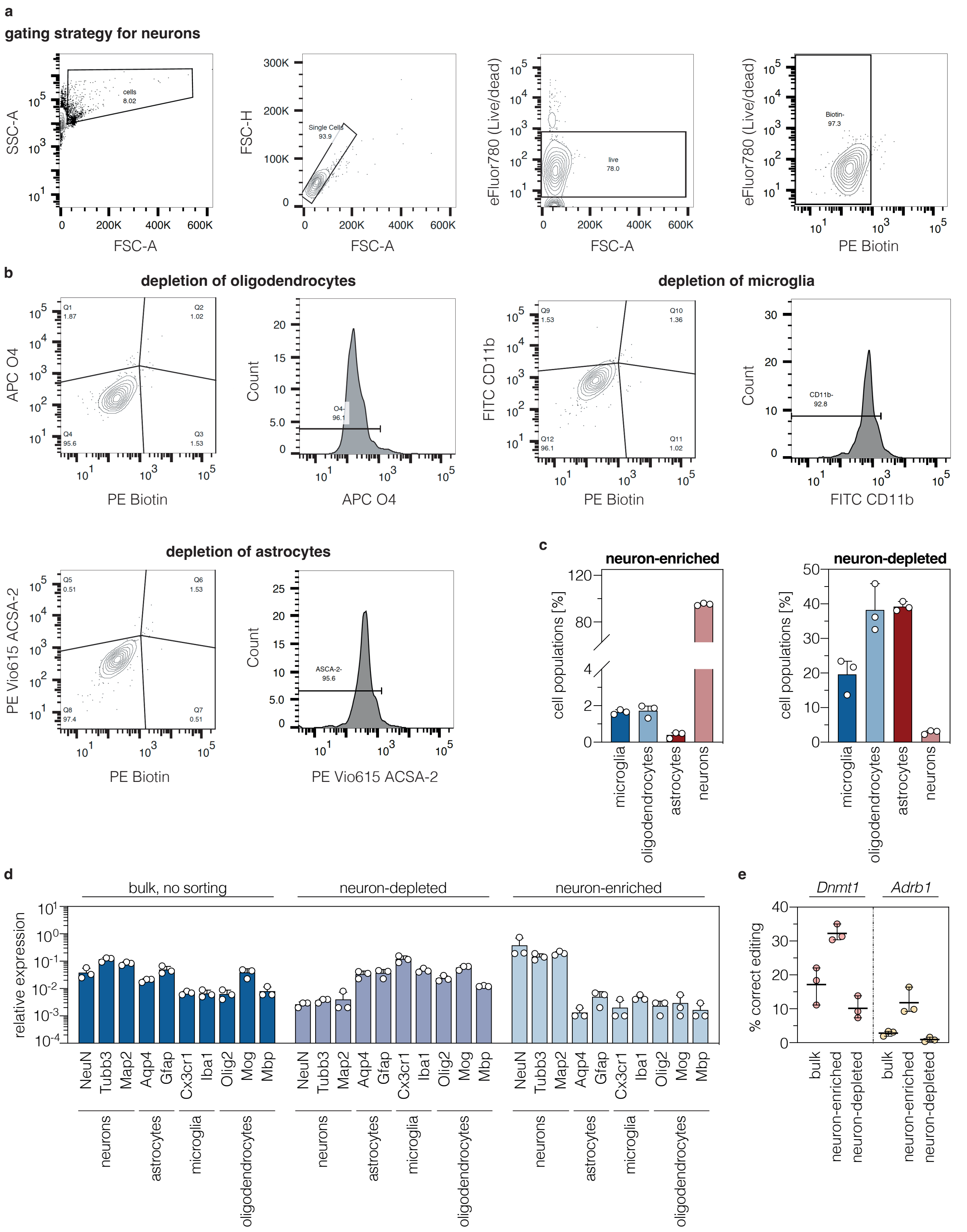

Supplementary figure 8 | Validation of MACS-purified neuronal populations. (**a-c**) Gating strategy for validation of oligodendrocyte-, microglia-, and astrocyte-depletion in neuron-enriched samples (a,b) and quantification of the respective populations (c). A negative selection for neuronal populations was applied during MACS. (**d**) Transcript levels of neuronal, astrocytic, microglial, and oligodendrocytic markers confirming the presence/absence of these populations in bulk, neuron-depleted, and neuron-enriched samples. Transcript levels were normalized to *Gapdh*. Bulk samples are defined as single-cell suspensions isolated from the cortex before magnetic sorting. (**e**) Editing rates of bulk, neuron-enriched, and neuron-depleted samples at the *Adrb1* and *Dnmt1* locus (*Adrb1*, AAV-PE3bmax-nT and AAV-W3-synth-cT;

Dnmt1, AAV-PEmax-nT and AAV-noW3-bGH-cT) at 24 weeks post-ICV injection. Data are represented as means±s.d. of 3 animals per locus.

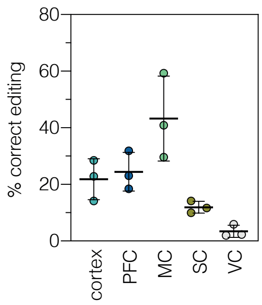

Supplementary figure 9 | Biodistribution of prime editing rates across cortical substructures. Comparison of prime editing rates of bulk cortex tissue and dissected cortical substructures. Animals were treated AAV-PE3bmax-nT and AAV-W3-synth-cT at P1 and tissues were harvested at 10 weeks post-ICV injection. Data are represented as means±s.d. of 3 animals. MC, motor cortex; PFC, prefrontal cortex; SC somatosensory cortex; VC, visual cortex.

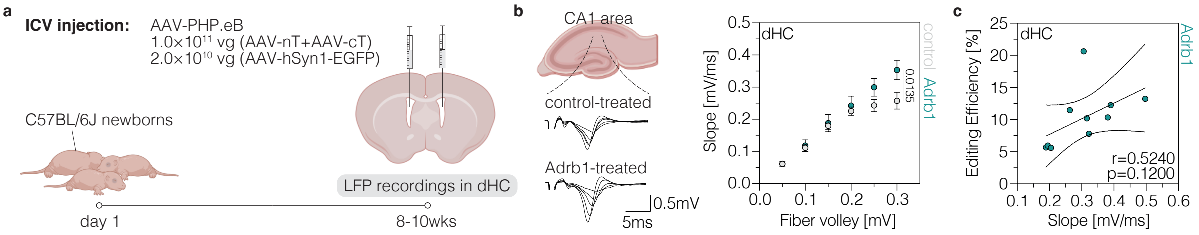

Supplementary figure 10 | Analysis of neuronal excitability in hippocampus slices of control- and Adrb1-treated mice. (**a**) Schematic depiction of the experimental setup and timeline. Created in BioRender (https://BioRender.com/7fw2d33). (**b**) Input-output curves for basal synaptic transmission in the area CA1 of the hippocampus. Schematic representation of the recording area (top left) and sample traces are shown for the output (fEPSP slope) at 0.05, 0.1, 0.15, 0.2, and 0.3 mV fiber volley amplitude (bottom left). (**c**) Pearson correlation of on-target editing and the slope at the 0.3mV fiber volley. Data are displayed as means±s.e.m. (control-treated, n=6 slices; Adrb1-treated, n=10 slices) and were analyzed using a two-way ANOVA with Bonferroni's multiple comparisons test. If not indicated, differences are not statistically significant (P>0.05). Correlation coefficient, P-value, and 95% confidence intervals are indicated in the respective plots. vg, vector genomes; nT/cT, N-/C-terminal PEmax AAV vector; hSyn1, human synapsin 1 promoter; LFP, local field potential; mV, millivolt; ms, millisecond; dHC, dorsal hippocampus.

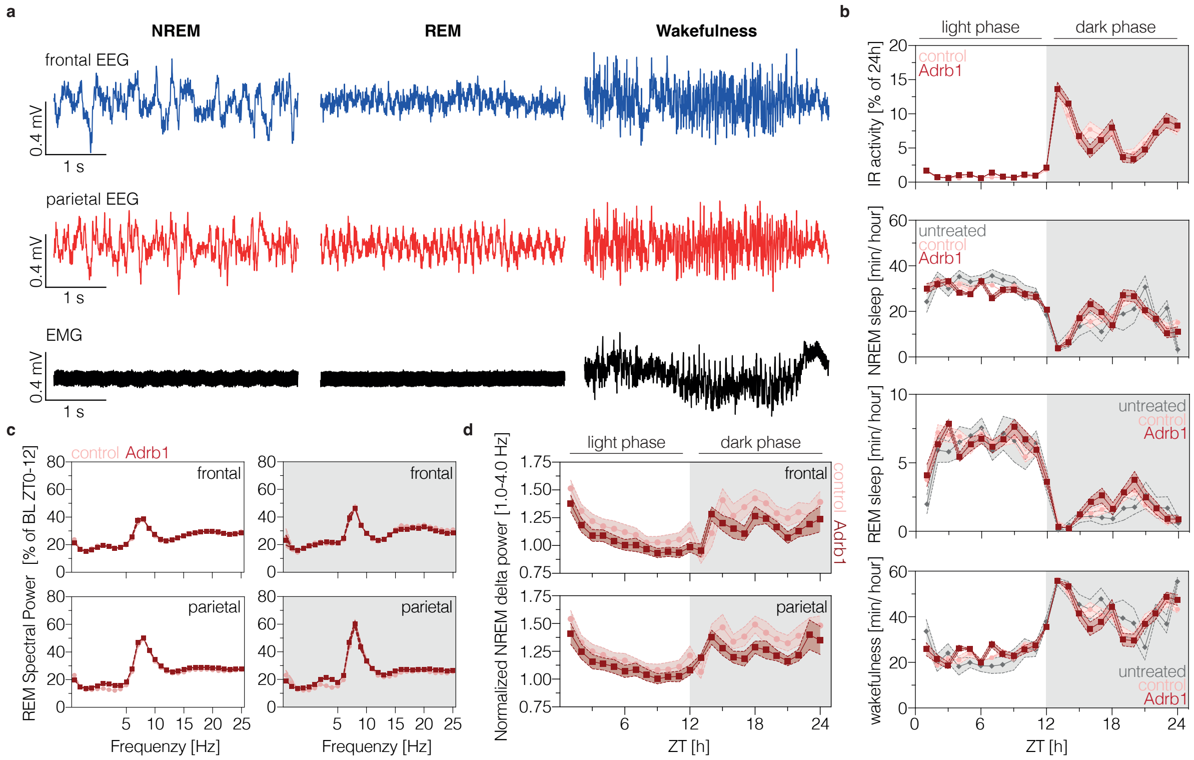

Supplementary figure 11 | Sleep EEG features of untreated, control-, and Adrb1-treated mice. (**a**) Representative examples of raw signals (n=12 mice per group) for NREM sleep, REM sleep, and wakefulness from the frontal EEG (top/blue), parietal EEG (middle/red), and EMG (bottom/black). (**b**) Quantifications of infrared (IR) activity (top), time spent (min/hour) in NREM sleep (middle top), REM sleep (middle bottom), and wakefulness (bottom), as an average over the recording days. (**c**) Normalized REM sleep EEG spectral power during the 12h light or dark phase as a percentage of the total power in all vigilance states. (**d**) Time course of SWA distribution across the day. Averages over the three recording days are shown. Areas highlighted in white indicate the light phase (ZT0-12) and areas highlighted in gray indicate the dark phase (ZT12-24). Control animals were treated with PEmax and an epegRNA targeting *Dnmt1*. Adrb1-treated animals were injected with PE3bmax and epegRNA1 targeting *Adrb1*. Untreated age-matched mice were used as additional controls. Data are displayed as means±s.e.m. (untreated, n=7 mice; control, n=12 mice; Adrb1, n=12 mice) and were analyzed using a mixed model two-way ANOVA with Geisser Greenhouse correction (b-d). If not indicated, differences are not statistically significant (P>0.05). mV, milliVolt; s, second; IR, infrared; min, minute; h, hour; ZT, zeitgeber; REM, rapid eye movement; NREM, non-rapid eye movement.

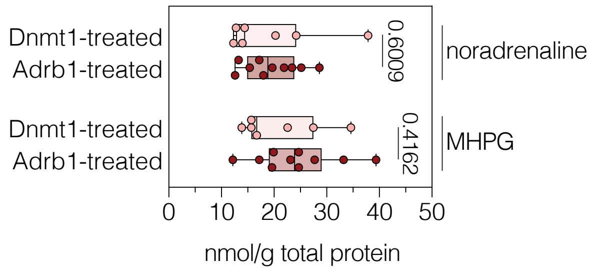

Supplementary figure 12 | Noradrenaline and MHPG levels in control- and Adrb1-treated brain tissues. *Adrb1*-expressing brain regions. UHPLC quantifications of noradrenaline (top) and its metabolite MHPG (3-methoxy-4-hydroxyphenylglycol, bottom) in brain tissues from control- (light pink) and Adrb1-treated (red) C57BL/6J mice. Control animals were treated with PEmax and an epegRNA targeting *Dnmt1*. Adrb1-treated animals were injected with PE3bmax and epegRNA1 targeting *Adrb1*. Data are displayed as means±s.d. (control, n=7 mice; Adrb1, n=10 mice) and were analyzed using a two-way ANOVA with Tukey's multiple comparisons test. g, gram.

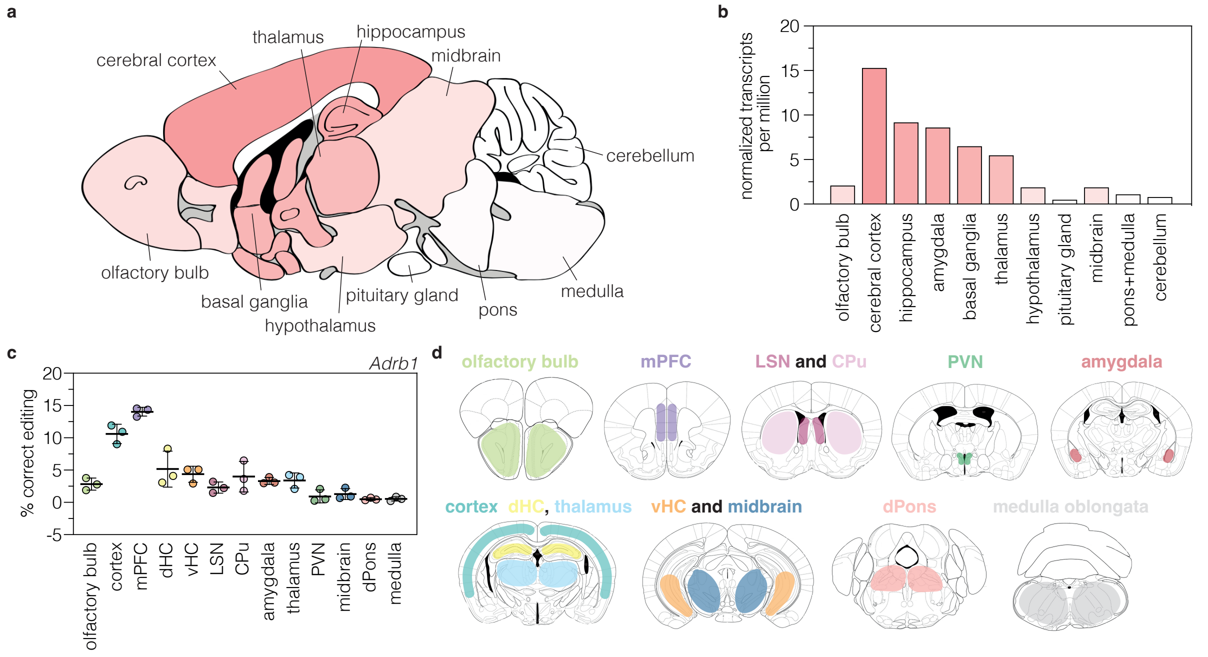

Supplementary figure 13 | Prime editing across *Adrb1*-expressing brain regions. (a,b) Color-coded *Adrb1*-expression (a) and normalized transcripts per million (nTPM, b) across the mouse brain (image credit: Human protein atlas[51]). Darker shades indicate higher expression. (**c**) Editing rates in brain regions with *Adrb1* expression (cut-off >1.0 nTPM in the mouse protein atlas[53]) in mice ICV-injected as newborns (n=3 mice; treated with AAV-PE3bmax-nT and AAV-W3-synth-cT). Each data point represents one animal. (**d**) The location of each region, isolated for deep sequencing, is indicated by color on the mouse brain atlas[81]. mPFC, medial prefrontal cortex; LSN, lateral septal nucleus; CPu, caudate putamen; PVN, paraventricular nucleus; d/vHC, dorsal/ventral hippocampus; dPons, dorsal pons. Color coding of isolated brain areas is identical in (c) and (d).

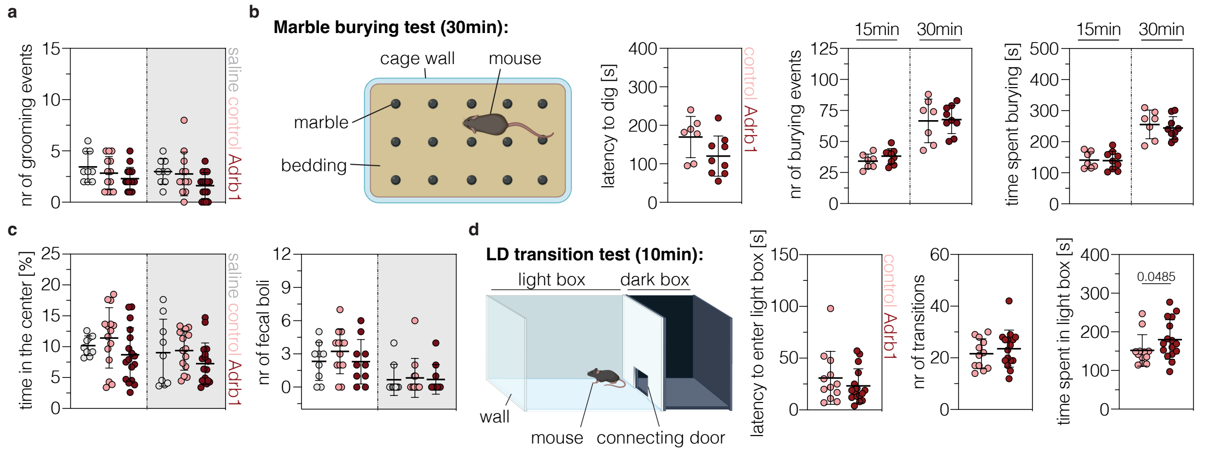

Supplementary figure 14 | Assessment of obsessive compulsive-like and anxiety-related behaviors. (**a,b**) Self-grooming events in the OF during the light and dark phase (a) and marble burying during the light phase (b) as indicators of obsessive compulsive-like behaviors in saline- (n=9), control- (n=7 or 13), or Adrb1-treated mice (n=9 or 12). Schematic representation of the marble burying test with the test duration in brackets is shown in (b). (**c**) Percentage of time spent in the center of the OF arena (left) and the number of fecal boli (right) as indicators of anxiety-related behaviors in saline- (n=8), control- (n=15), and Adrb1-treated mice (n=17). (**d**) Anxiety-related behaviors in the LD transition test for control- (n=12) and Adrb1-treated mice (n=16). All mice were kept in a 12:12 light/dark cycle. Areas highlighted in white indicate the light phase (ZT0-12) and areas highlighted in gray indicate the dark phase (ZT12-24). Control animals were treated with saline or PEmax and an epegRNA targeting *Dnmt1*. Adrb1-treated animals were treated with PE3bmax and epegRNA1 targeting *Adrb1*. Data are displayed as means±s.d. and were analyzed using a two-way ANOVA (a,c), an unpaired two-tailed Student's *t*-test (b, "latency to enter the light box [s]" and "nr of transitions" in d), or a Mann-Whitney test ("time spent in light box [s]" in d; *$P<0.05$). If not indicated, differences are not statistically significant ($P>0.05$). Each data point represents one animal. nr, number; min, minutes; s, seconds; LD, light-dark. Schematic representations in panels (b) and (d) were created in BioRender (https://BioRender.com/suhq26k).

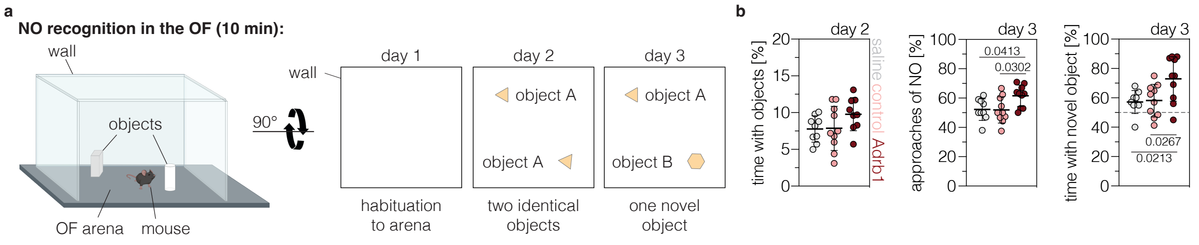

Supplementary figure 15 | Improved performance of Adrb1-treated mice in the NO recognition test. (**a**) Schematic representation of the NO recognition test. The duration of the test is indicated in brackets. Created in BioRender (https://BioRender.com/d1f8wci). (**b**) Percentage of time spent with the objects during the trial on day 2 (left), approaches of the NO on day 3 (middle), and time spent with the NO on day 3 (right) for saline- (n=9), control- (n=10), and Adrb1-treated mice (n=9) after 10 min (left) or 5 min (middle and right) exposure to the objects. Control animals were treated with saline or PEmax and an epegRNA targeting *Dnmt1*. Treated animals were injected with PE3bmax and epegRNA1 targeting *Adrb1*. The dashed line in (b, right) represents the 50% chance level. Statistical significance between each group and chance level was assessed using a one-sample t-test (saline, P=0.0239; control, P=0.0450; Adrb1, P=0.0010). Data are displayed as means±s.d. and were analyzed using a two-way ANOVA with Tukey's multiple comparisons test. If not indicated, differences are not statistically significant (P>0.05). Each data point represents one animal. NO, novel object; OF, open field.

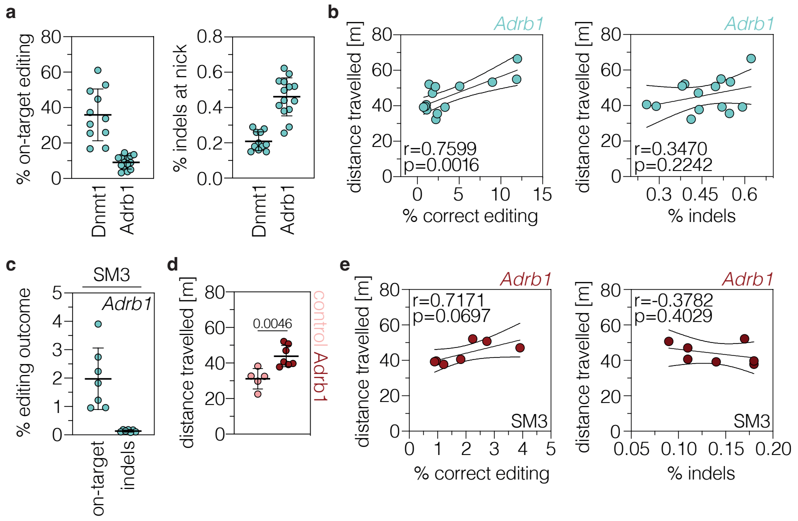

Supplementary figure 16 | On-target editing, but not indels, correlate with locomotor activity. (**a**) On-target editing (left) and indel rates at nicking site (right) in cortices of mice injected as newborns with either PEmax and an epegRNA targeting *Dnmt1* (n=11 mice) or PE3bmax and epegRNA1 targeting *Adrb1* (n=14 mice). (**b**) Pearson correlations of on-target editing (left) or indel formation (right) and distance traveled for Adrb1-treated animals (PE3bmax and epegRNA1; n=14 mice). (**c**) On-target editing and indel rates at the nicking site in cortices of mice injected as newborns with PEmax and epegRNA1-SM3 (SM3, n=7 mice). (**d**) Locomotor activity of Adrb1- (PEmax and epegRNA1-SM3; n=7) and control-treated (PEmax and epegRNA targeting *Dnmt1*; n=5) animals in the OF during the light phase. Data are displayed as means±s.d. and were analyzed using an unpaired two-tailed Student's *t*-test. (**e**) Pearson correlations of on-target editing (left) or indel formation (right) and distance traveled for Adrb1-treated animals (PEmax and epegRNA1-SM3; n=7 mice). Correlation coefficients, *P*-values, and 95% confidence intervals are indicated in the respective plots. Each data point represents one animal. SM, silent mutation.

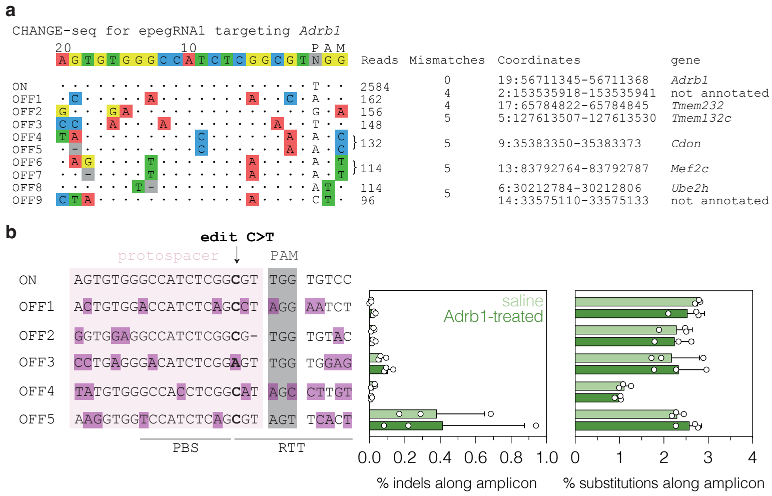

Supplementary figure 17 | Off-target editing in brains of saline- or Adrb1-treated mice. (**a**) Off-targets for the epegRNA1 protospacer targeting the *Adrb1* locus were experimentally identified using CHANGE-seq[50]. Off-targets with <90 reads or <3% of total reads are not shown. (**b**) Deep sequencing of the top 5 off-targets, identified by CHANGE-seq, in saline- or Adrb1-treated mice at 6 months post-ICV injection (>15'000 reads per site). The protospacer sequence (rose), mismatches (purple), PAM sequence (gray), and the position of the on-target edit (bold) are indicated. Indels and bystanders were quantified across the whole amplicon length. Data are displayed as means±s.d. of 3 animals per group and were analyzed using an unpaired two-tailed Student's t-test. If not indicated, differences are not significant (*P*>0.05).

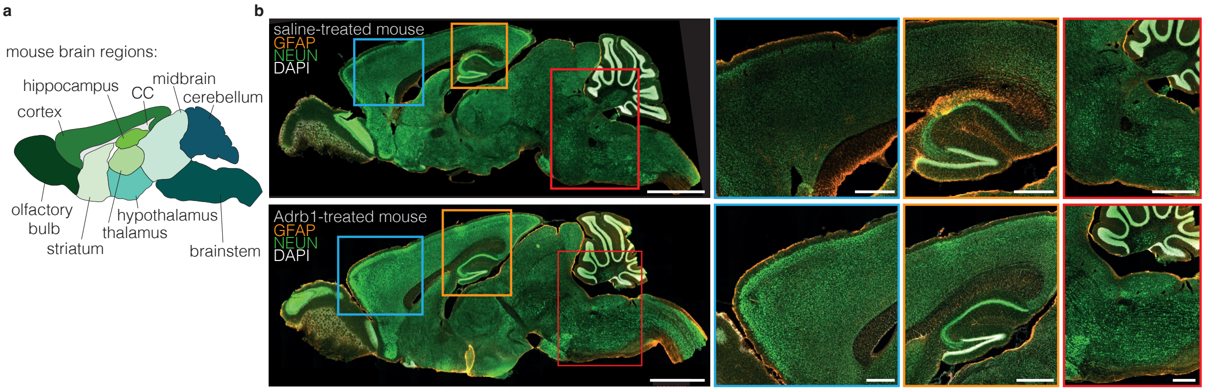

Supplementary figure 18 | Histological analysis of brain sections from saline- or Adrb1-treated mice. (**a**) Schematic representation of brain regions in a sagittal brain section. Regions are roughly depicted to scale. (**b**) Representative fluorescence micrographs of a sagittal brain section (n=2 sections per 3 mice) from saline- (top) or Adrb1-treated (bottom) mice, showing neuronal and astrocyte populations. Magnified images (top, right; bottom, right) are shown for selected regions. Scale bars (from left to right), 2000 µm; 500 µm; 500 µm; 1000 µm. HF, hippocampal formation.

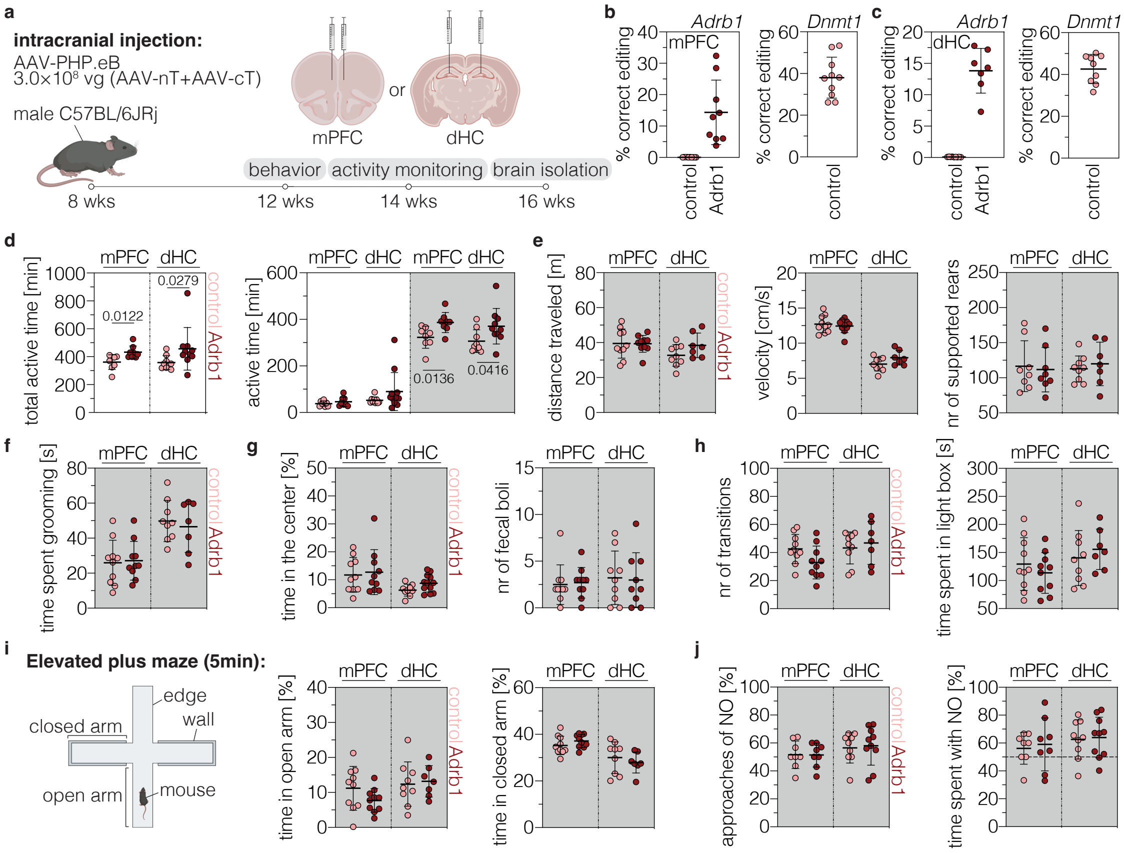

Supplementary figure 19 | Behavioral effect of the *Adrb1*^{A187V} mutation in the mPFC and dHC of adult mice. (**a**) Schematic depiction of the experimental setup for introducing the *Adrb1*^{A187V} mutation into the mPFC or dHC of adult male mice. (**b,c**) Editing efficiencies in mPFC (b; control, n=10; Adrb1, n=9) or dHC tissues (c; control, n=9; Adrb1, n=7) after intracranial delivery of prime editing components. (**d**) Comparison of IR activity in the home cage of control- and Adrb1-treated mice. (**e**) Locomotor activity, velocity, and number of wall-supported rears of control- (mPFC, n=8; dHC, n=8) and Adrb1-treated mice (mPFC, n=8; dHC, n=10) in the OF during the dark phase. (**f-i**) Quantification of obsessive compulsive-like behaviors in the OF (f) and anxiety-related behaviors in the OF (g), LD transition test (h), and elevated plus maze (i) for control- (mPFC, n=10; dHC, n=9) and Adrb1-treated mice (mPFC, n=10; dHC, n=7). Schematic representation of the elevated plus maze with the test duration in brackets is shown in (i). (**j**) Percentage of approaches of the NO on day 3 (left) and time spent with the NO on day 3 (right) after 5 min exposure to the objects (control: mPFC, n=8; dHC, n=9; Adrb1: mPFC, n=8; dHC, n=10). The horizontal dashed line in (j) represents the 50% chance level. Statistical significance between control- or Adrb1-treated groups and chance level were assessed using a one-sample t-test (mPFC, control/P<0.1636; Adrb1/P<0.2208; dHC, control/P<0.0269; Adrb1/P<0.0141). Areas highlighted in white indicate the light phase (ZT0-12) and areas highlighted in gray indicate the dark phase (ZT12-24). Control animals were treated with PEmax and an epegRNA targeting *Dnmt1*. Treated animals were injected with PE3bmax and epegRNA1 targeting *Adrb1*. Data are displayed as means±s.d. and were analyzed using an unpaired two-tailed Student's t-test (b,c,e-h; **P<0.05*) or an unpaired two-tailed Mann Whitney U test (d, "time in the center [%] for mPFC in g; "nr of fecal boli" for mPFC in g). If not indicated, differences are not significant (P>0.05). Each data point represents one animal. vg, vector genomes; nT/cT, N-/C-terminal AAV vector; wks, weeks; min, minute; m, meter; cm, centimeter; s, second; mPFC, medial prefrontal cortex; dHC, dorsal hippocampus; nr, number; NO, novel object. Schematic representations in panels (a) and (i) were created in BioRender (https://BioRender.com/utsy9wd).

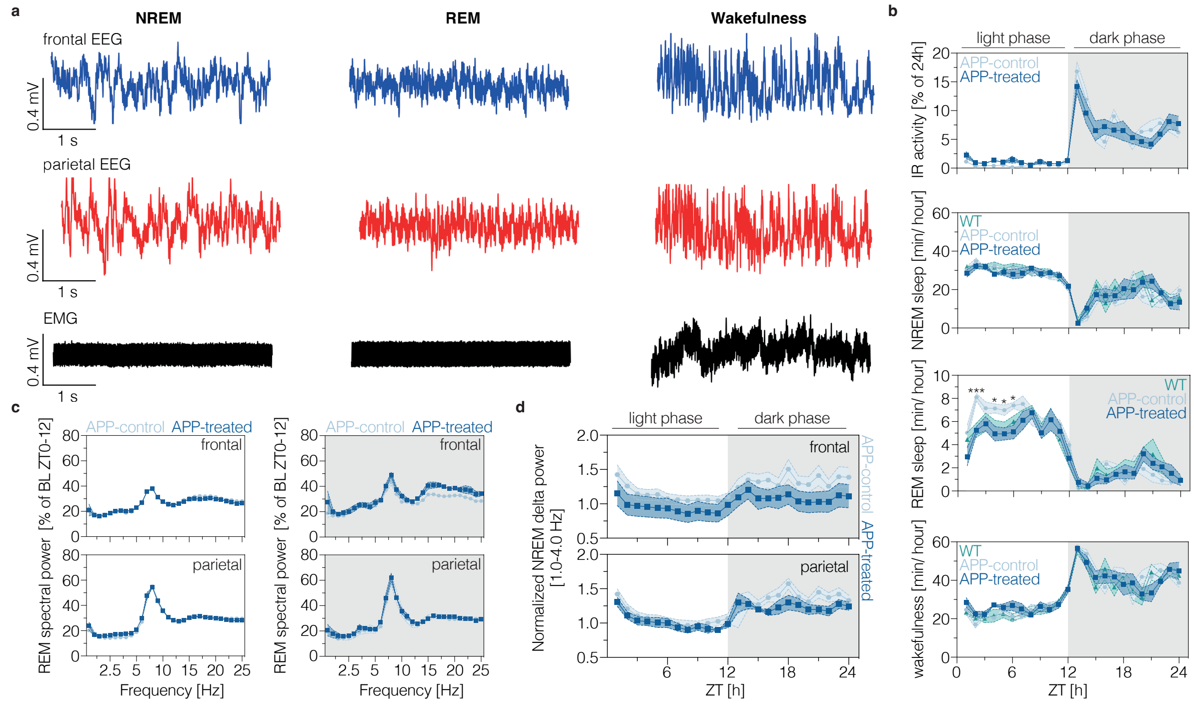

Supplementary figure 20 | Comparison of sleep EEG features in WT, APP-control, and APP-treated mice. (**a**) Representative examples of raw signals (n=6 mice per group) for NREM sleep, REM sleep, and wakefulness from the frontal EEG (top/blue), parietal EEG (middle/red), and EMG (bottom/black). (**b**) Quantifications of infrared (IR) activity (left), time spent (min/hour) in NREM sleep (middle left), REM sleep (middle right; ****P*=0.0001; **P*=0.0179; **P*=0.0167), and wakefulness (right), as an average over the three recording days. (**c**) Normalized REM sleep EEG spectral power during the 12h light or dark phase as a percentage of the total power in all vigilance states. (**d**) Time course of SWA distribution across the day. Averages over the three recording days are shown. Areas highlighted in white indicate the light phase (ZT0-12) and areas highlighted in gray indicate the dark phase (ZT12-24). WT and APP-control animals were treated with the N-terminal PEmax vector encoding the *Dnmt1* epegRNA expression cassette. APP-treated animals were injected with PE3bmax and epegRNA1 targeting *Adrb1*. Data are displayed as means±s.e.m. (n=6 mice per group) and were analyzed using a mixed model two-way ANOVA with Geisser Greenhouse correction followed by Šidák's multiple comparisons test (c-e). If not indicated, differences are not statistically significant (*P*>0.05). mV, milliVolt; s, second; IR, infrared; min, minute; h, hour; ZT, zeitgeber; REM, rapid eye movement; NREM, non-rapid eye movement.

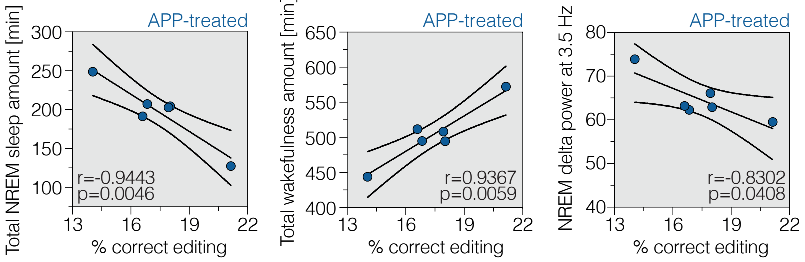

Supplementary figure 21 | Correlation of prime editing rates at the *Adrb1* locus and sleep EEG features in APP-treated mice. Pearson correlation of on-target prime editing frequency in whole cortex tissues and the total amount of NREM sleep (left), wakefulness (middle), or normalized NREM sleep delta power at 3.5 Hertz (Hz) during the dark phase (left; n=6 mice). Areas highlighted in gray indicate the dark phase. Correlation coefficients, *P*-values, and 95% confidence intervals are indicated in the respective plots. Each data point represents one animal (n=6 mice).

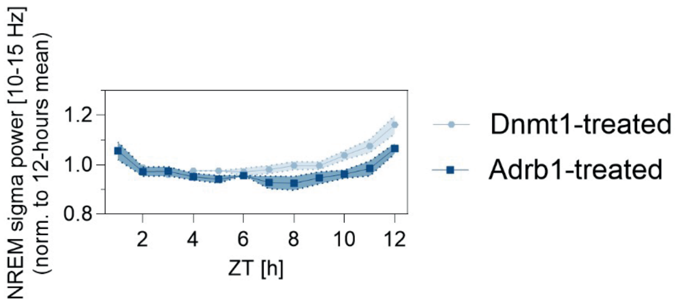

Supplementary figure 22 | Hourly NREM EEG σ power dynamics over the average light period of the three recording days in APP-control and APP-treated mice. NREM σ power is normalized to the average σ power over all 12-hours. Data are displayed as means±s.e.m. (n=6 mice per group) and were analyzed using a mixed model two-way ANOVA with Geisser Greenhouse correction (time x treatment; ***P*=0.003). Hz, Hertz; h, hour; ZT, zeitgeber.

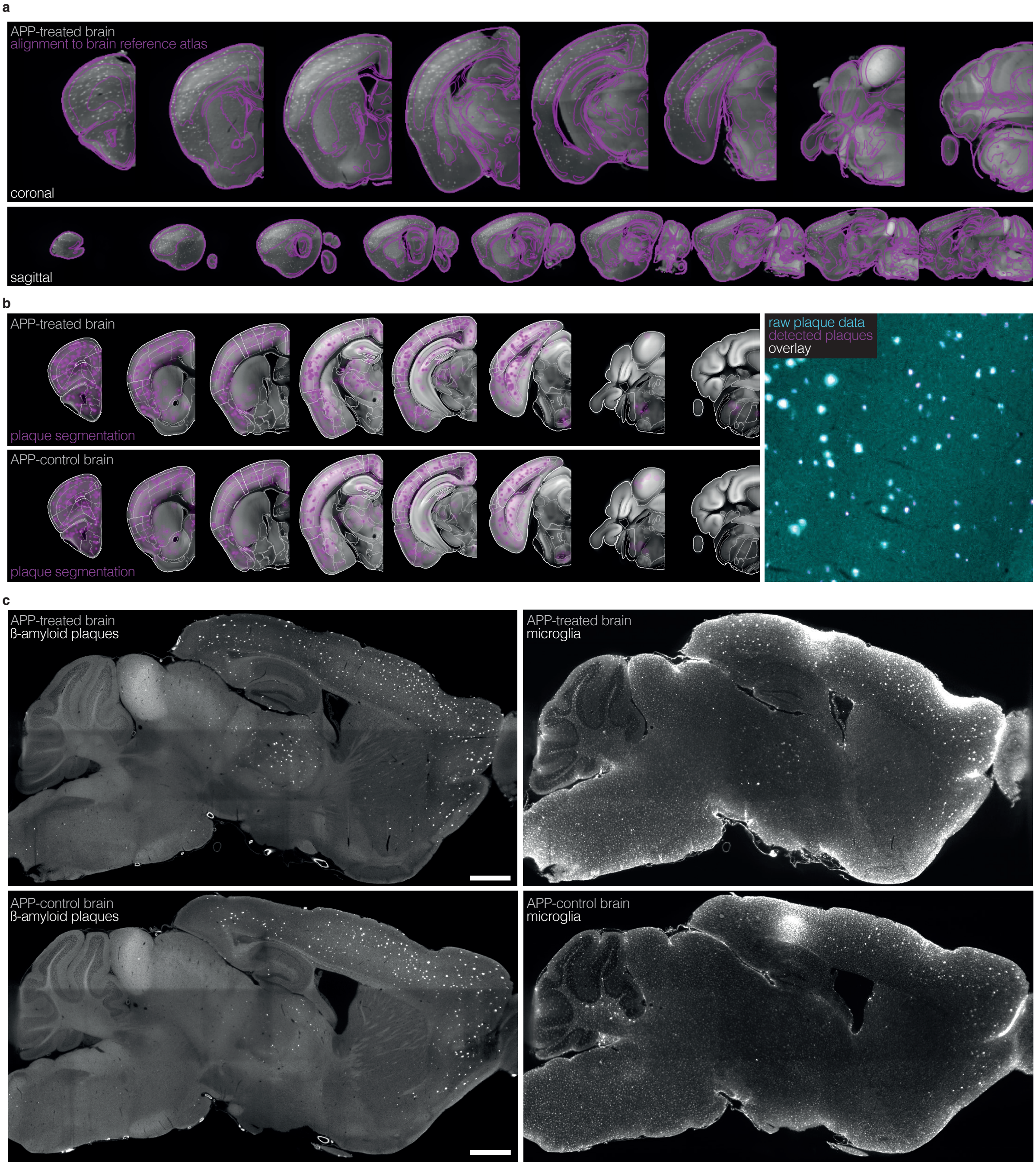

Supplementary figure 23 | Three-dimensional analysis of β-amyloid plaques and plaque-associated microglia in APP-control and APP-treated mice. (a) Alignment (purple) of coronal (top) and sagittal (bottom) fluorescence micrographs from cleared brain hemispheres to the Allen mouse brain reference atlas. **(b)** Segmentation (purple) of β-amyloid plaques in coronal fluorescence micrographs of cleared brain hemispheres (left, top and bottom). Representative image of the cortex (APP-control, n=7 brains; APP-treated, n=4 brains) showing the accuracy of the segmentation (purple) of β-amyloid plaques in the raw data (cyan). The shown density maps depict all voxels detected for β-amyloid plaques across the aligned data. **(c)** Representative brain sections (APP-control, n=7 brains; APP-treated, n=4 brains) revealing the regional distribution of β-amyloid plaques and microglia. WT and APP-control animals were treated with the N-terminal PEmax vector encoding the *Dnmt1* epegRNA expression cassette. APP-treated animals were injected with PE3bmax and epegRNA1 targeting *Adrb1*. Brains were isolated from APP-treated and APP-control mice at 17 weeks post-ICV injection. Scale bars, 200μm (c).

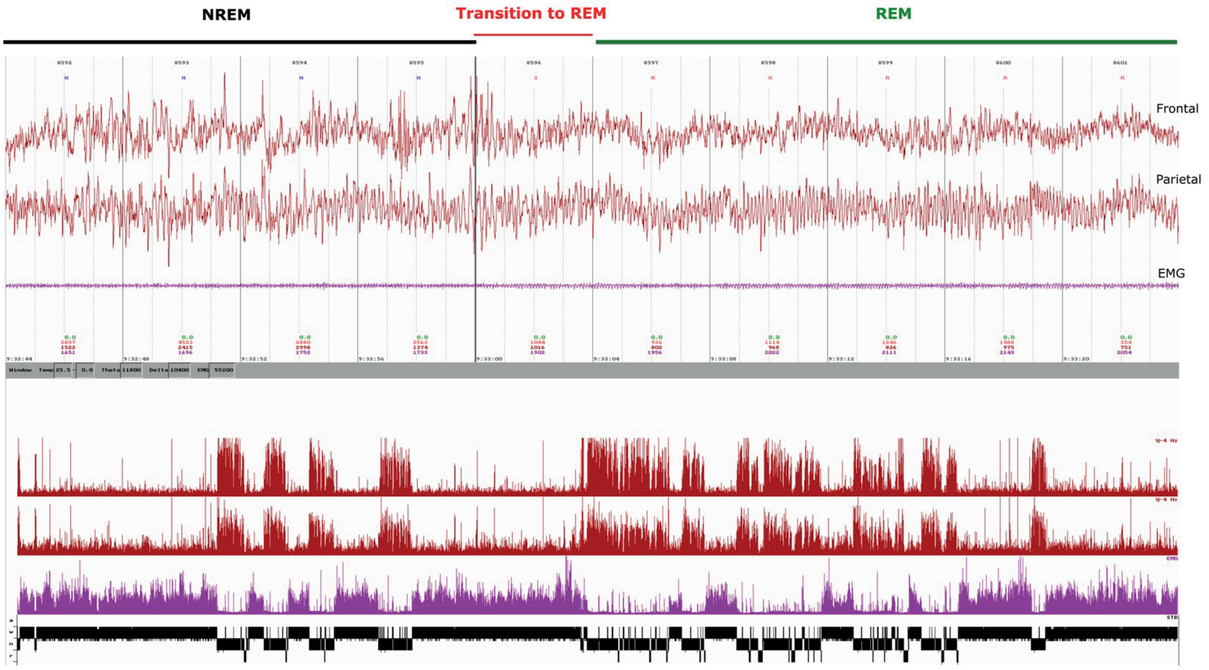

Supplementary figure 24 | Classification of "Transitions to REM sleep" in automated sleep recordings. Representative EEG traces (n=12 mice for control and Adrb1-treated groups each) of NREM and REM sleep as well as transition states from NREM to REM sleep indicated at the top. Control animals were treated with PEmax and an epegRNA targeting *Dnmt1*. Treated animals were injected with PE3bmax and epegRNA1 targeting *Adrb1*.

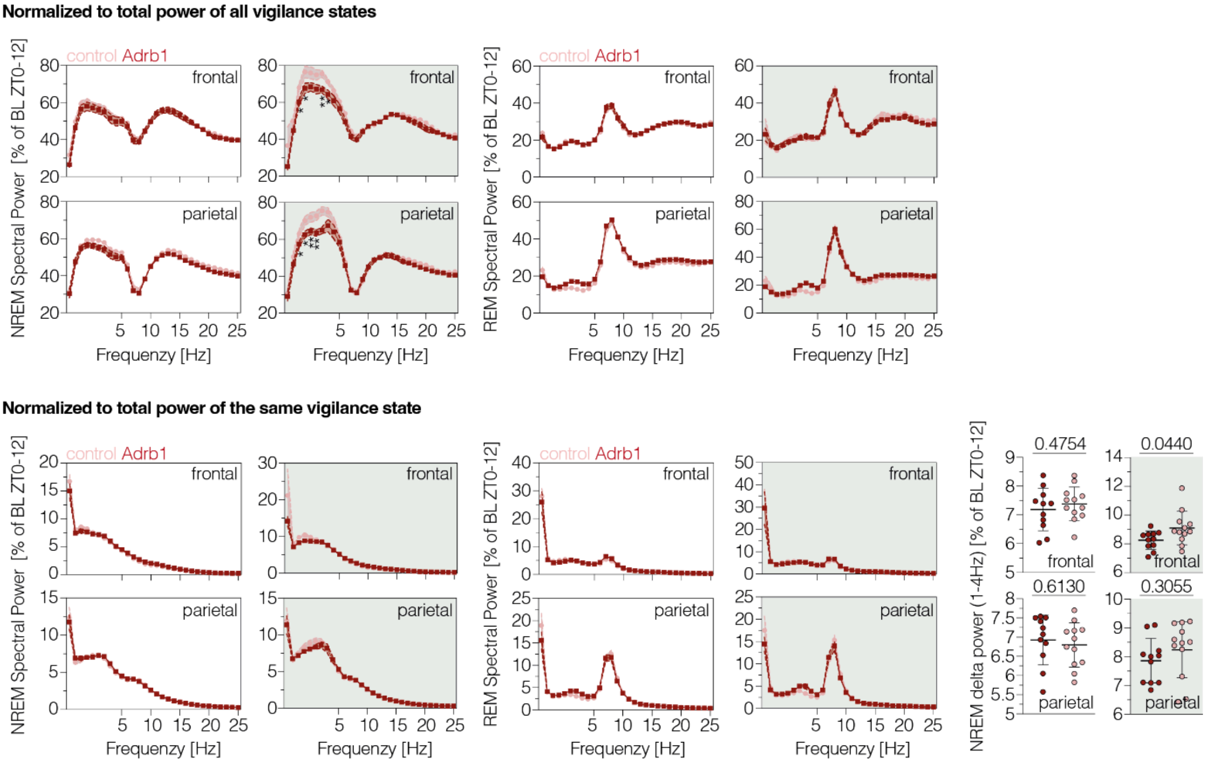

Supplementary figure 25 | Different normalization approaches for EEG spectral analysis. Comparison of NREM and REM power spectra after normalization to total power of all vigilance states (top) or total power of the same vigilance state (bottom). Data are displayed as means±s.e.m. and were analyzed using a mixed model two-way ANOVA with Geisser Greenhouse correction followed by Šidák's multiple comparisons test (top left: frontal, **P*=0.0217; **P*=0.0469; ***P*=0.0037; **P*=0.0237; parietal, **P*=0.0287; **P*=0.0469; ***P*=0.0071; ***P*=0.0018) or an unpaired two-tailed Student's t-test (bottom right). If not indicated, differences are not statistically significant (*P*>0.05). Areas highlighted in white indicate the light phase (ZT0-12) and areas highlighted in gray indicate the dark phase (ZT12-24). Control-treated animals were treated with PEmax and an epegRNA targeting *Dnmt1*. Adrb1-treated animals were injected with PE3bmax and epegRNA1 targeting *Adrb1*. ZT, zeitgeber time; REM, rapid eye movement; NREM, non-rapid eye movement; Hz, Hertz.

Supplementary tables

Supplementary table 1: Oligos used for cloning of PE and AAV plasmids.

oligo name	oligo sequence (5'→ 3')
N-intein_fwd	CAGATCCGCTAGAGATCCGCGGCCGCTAATACGACTCACTATAGGGAGAGCC
C-intein_rev	TCCTCTTCTTCTTGGGCTCGAATTCGCTGCCGTCGGCGGTTCT
split867_fwd	GCAGCGGTGGCGGCGGCAGCAGCGACAACGTGCCCTCC
split866_rev	CCGCCACCGCTGCCGCCGCCACCCTTGCCCCGGTTCTTGTCG
split1025_fwd	GCAGCGGTGGCGGCGGCAGCAGCGAGCAGGAAATCGGCAAG
split1024_rev	CCACCGCTGCCGCCGCCACCCTTGGCGATCATCTTCCG
split573_fwd	GCAGCGGTGGCGGCGGCAGCTGCTTCGACTCCGTGGAAATCTCC
split572_rev	CCGCCACCGCTGCCGCCGCCACCCTCGATTTTCTTGAAGTAGTCCTC
split713_fwd	GCAGCGGTGGCGGCGGCAGCTCCGGCCAGGGCGATA
split712_rev	CCGCCACCGCTGCCGCCGCCACCCACCTGGGCTTTCTGGATGT
AAV-BPNLS-PEmax712_fwd	GAGCGCAGTCGAGAGTTGGACCGGTGCCACCATGAAACGGACAG
AAV-BPNLS-PEmax713_rev	TCTGTTGGCGAAGCCGTCGGACTTCAGGAAATCCAGGATTG
AAV-BPNLS-Nintein_fwd	AAGCGGAAAGTCGACAAGAAGTACAGCATC
AAV-BPNLS-Nintein_rev	CGCCAGAATTAGGCAGGTTATCCACTCTCA
AAV-pegRNA-optscf_fwd	ACTCCATCACTAGGGGTTCCTGCGGCCGCGAGGGCCTATTTCCCATG
AAV-pegRNA-optscf_rev	GATGCGGTGGGCTCTATGGCTCGAGAAAAAAAGCCATCTCGGTG
AAV-pegRNAevo-optscf_rev	GATGCGGTGGGCTCTATGGCTCGAGAAAAAAATTCTAGTTGGTTTAACGC
AAV-3bpegRNAevo-optscf_rev	ATCATGGGAAATAGGCCCTCGAATTCAAAAAAATTCTAGTTGGTTTAACGC
AAV-3bpegRNAevo-optscf_fwd	AAACCAACTAGAATTTTTTTGAATTCGAGGGCCTATTTCCCATG
AAV-3bngRNA-optscf_rev	GATGCGGTGGGCTCTATGGCTCGAGAAAAAAAAGCACCGACTC
AAV-3bngRNAevo-optscf_rev	GATGCGGTGGGCTCTATGGCTCGAGAAAAAAAAATTCTAGTTGGTTTAACGC
pegRNA-U6-NdeI_fwd	TGTTTTAAAATGGACTATCATATGCTTACCGTAACTTGAAAGTATTTC
AAV-bGHpolyA_fwd	GATGCGGTGGGCTCTATGG
AAV-U6_rev	GGCCGCGAGGGCCTATTT

Supplementary table 2: Oligos used for cloning of pegRNA and nicking sgRNA plasmids.

oligo name	oligo sequence (5'→ 3')
pegRNA1-Adrb1-spacer_fwd	CACCGAGTGTGGGCCATCTCGGCGTGTTTT
pegRNA1-Adrb1-spacer_rev	CTCTAAAACACGCCGAGATGGCCCACACTC
pegRNA1.1-Adrb1-ext_fwd	GTGCGGACACCAACACCGAGATGGC
pegRNA1.1-Adrb1-ext_rev	AAAAGCCATCTCGGTGTTGGTGTCC
pegRNA1.2-Adrb1-ext_fwd	GTGCAAGGACACCAACACCGAGATGGC
pegRNA1.2-Adrb1-ext_rev	AAAAGCCATCTCGGTGTTGGTGTCCTT
pegRNA1.3-Adrb1-ext_fwd	GTGCGGAAGGACACCAACACCGAGATGGC
pegRNA1.3-Adrb1-ext_rev	AAAAGCCATCTCGGTGTTGGTGTCCTTCC
pegRNA1.4-Adrb1-ext_fwd	GTGCGGACACCAACACCGAGATGGCCCA
pegRNA1.4-Adrb1-ext_rev	AAAATGGGCCATCTCGGTGTTGGTGTCC
pegRNA1.5-Adrb1-ext_fwd	GTGCAAGGACACCAACACCGAGATGGCCCA
pegRNA1.5-Adrb1-ext_rev	AAAATGGGCCATCTCGGTGTTGGTGTCCTT
pegRNA1.6-Adrb1-ext_fwd	GTGCGGAAGGACACCAACACCGAGATGGCCCA
pegRNA1.6-Adrb1-ext_rev	AAAATGGGCCATCTCGGTGTTGGTGTCCTTCC
pegRNA-scaffold_fwd	AGAGCTAGAAATAGCAAGTTAAAATAAGGCTAGTCCGTTATCAACTTGAAAAAGTGGCACCGAGTCG
pegRNA-scaffold_rev	GCACCGACTCGGTGCCACTTTTTCAAGTTGATAACGGACTAGCCTTATTTTAACTTGCTATTTCTAG
GG-epegRNA1-spacer_fwd	CACCGAGTGTGGGCCATCTCGGCGTGTTTC
GG-epegRNA1-spacer_rev	CTCTGAAACACGCCGAGATGGCCCACACTC
GG-scaffold-optscf_fwd	AGAGCTATGCTGGAAACAGCATAGCAAGTTGAAATAAGGCTAGTCCGTTATCAACTTGAAAAAGTGGCACCGAGTCG
GG-scaffold-optscf_rev	GCACCGACTCGGTGCCACTTTTTCAAGTTGATAACGGACTAGCCTTATTTCAACTTGCTATGCTGTTTCCAGCATAG
GG-evoRT/PBS-SM1_fwd	GTGCGGACACCAGCACCGAGATGGC
GG-evoRT/PBS-SM1_rev	CGCGGCCATCTCGGTGCTGGTGTCC
GG-evoRT/PBS-SM2_fwd	GTGCGGACACTAACACCGAGATGGC
GG-evoRT/PBS-SM2_rev	CGCGGCCATCTCGGTGTTAGTGTCC
GG-evoRT/PBS-SM3_fwd	GTGCGGACACAAGCACCGAGATGGC
GG-evoRT/PBS-SM3_rev	CGCGGCCATCTCGGTGCTTGTGTCC
GG-evoRT/PBS-SM4_fwd	GTGCGGACACGAGCACCGAGATGGC

Supplementary table 2 continued.	
oligo name	oligo sequence (5'→ 3')
GG-evoRT/PBS-SM4_rev	CGCGGCCATCTCGGTGCTCGTGTCC
GG-evoRT/PBS-SM5_fwd	GTGCGGACACTAGCACCGAGATGGC
GG-evoRT/PBS-SM5_rev	CGCGGCCATCTCGGTGCTAGTGTCC
PE3ngRNA3.1_fwd	CCACGGCCCACACTGTGCACACGA
PE3ngRNA3.1_rev	AAACTCGTGTGCACAGTGTGGGCC
PE3ngRNA3.2_fwd	CACCGAAGGACACCAACGCCGAGA
PE3ngRNA3.2_rev	AAACTCTCGGCGTTGGTGTCCTTC
PE3ngRNA3.3_fwd	CCACGCGACGTGATGGCGAGGTAG
PE3ngRNA3.3_rev	AAACCTACCTCGCCATCACGTCGC
PE3ngRNA3.4_fwd	CCACGCGCGCGTCAGCAAACTC
PE3ngRNA3.4_rev	AAACGAGTTTGCTGACGCGCGCGC
U6-epegRNAs_fwd	TGTGGAAAGGACGAAACACC
GG-epeg-tmpknot_rev	CAGGGGGGGGCTCCTGACCCTGTACTGTGTGCAGTGGaAG
GG-epeg-evopreQ1_rev	TAACGCGTAACTAGATAGAACCGCGTGTACTGTGTGCAGTGGaAG
Dnmt1_spacer-fwd	caccGCGGGCTGGAGCTGTTCGCGCgtttt
Dnmt1_spacer-rev	ctctaaaacGCCTAATGTACTGTGTGCAGc
Dnmt1_RT/PBS-fwd	gtgcAAGATGgCAGCGCGAACAGCTCCAG
Dnmt1_RT/PBS-rev	aaaaCTGGAGCTGTTCGCGCTGCCATCTT
Adrb1-epeg-tmpknot_rev	CAGGGGGGGGCTCCTGACCCGCCATCTCGGTGTTGGTGTC
Adrb1-epeg-evopreQ1_rev	TAACGCGTAACTAGATAGAACCGCGGCCATCTCGGTGTTGGTGTC
PE3bngRNA-Adrb1-tmp_rev	CAGGGGGGGGCTCCTGACCCGCACCGACTCGGTGCCACTT
PE3bngRNA-Adrb1-evo_rev	TAACGCGTAACTAGATAGAACCGCGGCACCGACTCGGTGCCACTT
pegRNA-eIF2B4_spacer_fwd	CACCGAGACCAGATTCAACA ACCAGGTTTT
pegRNA-eIF2B4_spacer_rev	CTCTAAAACCTGGTTGTTGAATCTGGTCTC
pegRNA-eIF2B4_RT/PBS_fwd	GTGCGTCCCTCCGGTTGTTGAATCTG
pegRNA-eIF2B4_RT/PBS_rev	AAAACAGATTCAACAACCGGAGGGAC
evo-eIF2B4_rev	CCCAAGCTTAAAAAAATTCTAGTTGGTTTAACGCGTAACTAGATAGAA CCGCGCAGATTCAACAACCGGA
PE3ngRNA-eIF2B4_fwd	CACCGCAGTGTAAGCGGGGAGACC
PE3ngRNA-eIF2B4_rev	AAACGGTCTCCCCGCTTACACTGC
evo-ngRNAs_rev	ACGCCAAGCTTAAAAAAATTCTAGTTGGTTTAACGCGTAACTAGATAGA ACCGCGGCACCGACTCGGTGC
pegRNA-eIF2B5_spacer_fwd	CACCGCCATCACCACGTTGTCCTCAGTTTT
pegRNA-eIF2B5_spacer_rev	CTCTAAAACTGAGGACAACGTGGTGATGGC
pegRNA-eIF2B5_RT/PBS_fwd	GTGCACACGCTGCCATGAGGACAACGT
pegRNA-eIF2B5_RT/PBS_rev	AAAAACGTTGTCCTCATGGCAGCGTGT
evo-eIF2B5_rev	CCCAAGCTTAAAAAAATTCTAGTTGGTTTAACGCGTAACTAGATAGAA CCGCGACGTTGTCCTCATGGC
PE3ngRNA-eIF2B5_fwd	CACCGAAATGTCTCTGTGATGACAA
PE3ngRNA-eIF2B5_rev	AAACTTGTCATCACAGAGACATTTC
pegRNA-otc_spacer_fwd	CACCGACCACACAAGACATTCACTTGTTTT
pegRNA-otc_spacer_rev	CTCTAAAACAAGTGAATGTCTTGTGTGGTC
pegRNA-otc_RT/PBS_fwd	GTGCACCGAGCGGTGTCTGTGAGACTTTCATTCACACCCAAGTGAATG TCTTGTG
pegRNA-otc_RT/PBS_rev	AAAACACAAGACATTCACTTGGGTGTGAATGAAAGTCTCACAGACACC GCTCGGT
evo-otc_rev	CCCAAGCTTAAAAAAATTCTAGTTGGTTTAACGCGTAACTAGATAGAA CCGCGCACAAGACATTCACTTGGGT
PE3ngRNA-otc_fwd	CACCGACCACACAAGACATTCACTT
PE3ngRNA-otc_rev	AAACAAGTGAATGTCTTGTGTGGTC

Supplementary table 3: Oligos used for RT-qPCR.

oligo name	oligo sequence (5'→ 3')
RTqPCR-mAdrb1_fwd	GCTCTGGACTTCGGTAGATGTG
RTqPCR-mAdrb1_rev	CGTCAGCAAACTCTGGTAGCGA
RTqPCR-hAdrb1_fwd	TTCCTGCCCATCCTCATGCACT
RTqPCR-hAdrb1_rev	GTAGAAGGAGACTACGGACGAG
RTqPCR-hGAPDH_fwd	GTCTCCTCTGACTTCAACAGCG
RTqPCR-hGAPDH_rev	ACCACCCTGTTGCTGTAGCCAA
RTqPCR-mGAPDH_fwd	CATCACTGCCACCCAGAAGACTG
RTqPCR-mGAPDH_rev	ATGCCAGTGAGCTTCCCGTTCAG
RTqPCR-NeuN_fwd	CACCACTCTCTTGTCCGTTTGC
RTqPCR-NeuN_rev	GGCTGAGCATATCTGTAAGCTGC
RTqPCR-Tubb3_fwd	CATCAGCGATGAGCACGGCATA
RTqPCR-Tubb3_rev	GGTTCCAAGTCCACCAGAATGG
RTqPCR-Map2_fwd	GCTGTAGCAGTCCTGAAAGGTG
RTqPCR-Map2_rev	CTTCCTCCACTGTGGCTGTTTG

Supplementary table 3 continued.

oligo name	oligo sequence (5'→ 3')
RTqPCR-Aqp4_fwd	AGCCAGCATGAATCCAGCTCGA
RTqPCR-Aqp4_rev	TCATAAAGGGCACCTGCCAGCA
RTqPCR-Gfap_fwd	ACATCGAGATCGCCACCTACA
RTqPCR-Gfap_rev	CCACGATGTTCCTCTTGAGGTG
RTqPCR-Iba1_fwd	TCTGCCGTCCAAACTTGAAGCC
RTqPCR-Iba1_rev	CTCTTCAGCTCTAGGTGGGTCT
RTqPCR-Cx3cr1_fwd	GAGCATCACTGACATCTACCTCC
RTqPCR-Cx3cr1_rev	AGAAGGCAGTCGTGAGCTTGCA
RTqPCR-Olig2_fwd	ATGCACGACCTCAACATCGCCA
RTqPCR-Olig2_rev	ACCAGTCGCTTCATCTCCTCCA
RTqPCR-Mog_fwd	GATGAAGGAGGCTACACCTGCT
RTqPCR-Mog_rev	CGTAGGCACAAGTGCGATGAGA
RTqPCR-Mbp_fwd	ATTCACCGAGGAGAGGCTGGAA
RTqPCR-Mbp_rev	TGTGTGCTTGGAGTCTGTCACC

Supplementary table 4: List of antibodies used in this study.

antibody	clone	host species	dilution	application
FITC anti-CD11b	M1/70	rat	1:50	FACS
PE Biotin	Bio3-18E7	mouse	1:50	FACS
PE-Vio®615 ACSA-2	REA969	rec-human	1:50	FACS
APC-O4	REA576	rec-human	1:50	FACS
NEUN	ab104224	mouse	1:500	histology
GFAP	ab95231	chicken	1:1'500	histology
Iba1	019-19741	rabbit	1:1'000	tissue clearing, primary
anti-chicken Cy5	JIR-703-175-155	donkey	1:500	histology, secondary
anti-mouse Cy3	JIR-715-165-151	donkey	1:500	histology, secondary
anti-rabbit 647	A-31573	donkey	1:400	tissue clearing, secondary

Supplementary table 5: Oligos used for deep sequencing.

Oligo name	oligo sequence (5'→ 3')
HTS-Adrb1-endogenous_fwd	CTTTCCCTACACGACGCTCTTCCGATCTNNNNNNCCAGCATTGAGACCCTGTGT
HTS-Adrb1-endogenous_rev	GGAGTTCAGACGTGTGCTCTTCCGATCTNNNNNNCATGAGGATGGGCAGGAAGG
HTS-Adrb1-PiggyBac_rev	GGAGTTCAGACGTGTGCTCTTCCGATCTNNNNNNATAGGGCCCTCTAGACGCTT
HTS-Dnmt1-invivo_fwd	CTTTCCCTACACGACGCTCTTCCGATCTNNNNNNGTCTTCCCCCACTCTCTTGC
HTS-Dnmt1-invivo_rev	GGAGTTCAGACGTGTGCTCTTCCGATCTNNNNNNCCCCAATATATGCCTCGGC
HTS-Adrb1-invivo_fwd	CTTTCCCTACACGACGCTCTTCCGATCTNNNNNNTCGCTACCAGAGTTTGCTGA
HTS-Adrb1-invivo_rev	GGAGTTCAGACGTGTGCTCTTCCGATCTNNNNNNAGCACTTGGGGTCGTTGTAG
HTS-eIF2B4-PiggyBac_fwd	CTTTCCCTACACGACGCTCTTCCGATCTNNNNNNGTGCTGGAATTCCTAAAAGG
HTS-eIF2B4-PiggyBac_rev	GGAGTTCAGACGTGTGCTCTTCCGATCTNNNNNNGTGATCACCAGATCCACGAG
HTS-eIF2B5-PiggyBac_fwd	CTTTCCCTACACGACGCTCTTCCGATCTNNNNNNGATCACAGAGTTGGGCAGAC
HTS-eIF2B5-PiggyBac_rev	GGAGTTCAGACGTGTGCTCTTCCGATCTNNNNNNCCTTGGGTCTTCTGGAAGTG
HTS-Otc-endogenous_fwd	CTTTCCCTACACGACGCTCTTCCGATCTNNNNNNGGGAGGACACCCTTCCTTTC
HTS-Otc-PiggyBac_rev	GGAGTTCAGACGTGTGCTCTTCCGATCTNNNNNNCAGTCCCTACCTGTGCCCAC

Supplementary table 6: Nucleotide sequences of HTS amplicons.

amplicon name	amplicon sequence (5’→ 3’)
Adrb1	CCAGCATTGAGACCCTGTGTGTCATCGCCCTGGACCGCTACCTCGCCATCACGTCGCC<br>CTTTCGCTACCAGAGTTTGCTGACGCGCGCGCGAGCGCGGGCCCTCGTGTGCACAGT<br>GTGGGCCATCTCGGCGTTGGTGTCCTTCCTGCCCATCCTCATG
Dnmt1	GTCTTCCCCCACTCTCTTGCCCTGTGTGGTACATGCTGCTTCCGCTTGCGCCGCCCCCT<br>CCCAATTGGTTTCCGCGCGCGCGAAAAAGCCGGGGTCTCGTTCAGAGCTGTTCTGTCG<br>TCTGCAACCTGCAAGATGCCAGCGCGAACAGCTCCAGCCCGAGTGCCTGCGCTTGCC<br>TCCCCGGCAGGCTCGCTCCCGGACCATGTCCGCAGGCGGTAGGTGCCACGCAGGGTG<br>GGGGTGAGGGGCGGGACCGATGCCGAGGCATATATTGGGGG
EIF2B4	GTGCTGGAATTCCTAAAAGGATGTTGACACCCACTTGTCATTTCAGATGATCCCGATG<br>ATCTGCAGTGTAAGCGGGGAGACCAGGTGGCCCTGGCTAACTGGCAGAGCCACCCGT<br>CCCTCTGGTTGTTGAATCTGGTCTATGACGTGACTCCACCTGAGCTCGTGGATCTGGT<br>GATCAC
EIF2B5	GATCACAGAGTTGGGCAGACTAACTGTGCCTCTGGTTCTTAATAGGTTAAGAAGGAA<br>GCTAGAAAAAAATGTCTCTGTGATGACAATGGTCTTCAAAGAGTCGTCACCCAGCCA<br>CCCTACACACTGCCATGAGGACAACGTGGTGATGGCTGTGGACAGCGCCACCAACAG<br>GGTTCTTCACTTCCAGAAGACCCAAGG
Otc	GGGAGGACACCCTTCCTTTCTTACCACACAAGACATTCACTTGGGTGTGAATGAAAGT<br>CTCACAGACACCGCTCAGTTTGTAAAACTTTTCTTCCTTCCAAAGTTTATTTCAAACTC<br>TGATGGGTTAGTTTAAAAGAGAAGATGATGCTTCTCCTTAGATAATGGTCTCCCCGGG<br>TGGGCACAGGTAGGGACTG

Supplementary table 7: Amino acid sequences of intein-split PEmax p.713 and p.714 constructs.

Intein-split PEmax p.713: p.NLS/n*Sp*Cas9$^{1-713}$(R221K,N394K)/linker/N-intein/NLS
MKRTADGSEFESPKKKRKVDKKYSIGLDIGTNSVGWAVITDEYKVPSKKFKVLGNTDRHSIKKNLIG<br>ALLFDSGETAEATRLKRTARRRYTRRKNRICYLQEIFSNEMAKVDDSFFHRLEESFLVEEDKKHERH<br>PIFGNIVDEVAYHEKYPTIYHLRKKLVDSTDKADLRLIYLALAHMIKFRGHFLIEGDLNPDNSDVDKL<br>FIQLVQTYNQLFEENPINASGVDAKAILSARLSKSRKLENLIAQLPGEKKNGLFGNLIALSLGLTPNFK<br>SNFDLAEDAKLQLSKDTYDDDLDNLLAQIGDQYADLFLAAKNLSDAILLSDILRVNTEITKAPLSAS<br>MIKRYDEHHQDLTLLKALVRQQLPEKYKEIFFDQSKNGYAGYIDGGASQEEFYKFIKPILEKMDGTE<br>ELLVKLKREDLLRKQRTFDNGSIPHQIHLGELHAILRRQEDFYPFLKDNREKIEKILTFRIPYYVGPLA<br>RGNSRFAWMTRKSEETITPWNFEEVVDKGASAQSFIERMTNFDKNLPNEKVLPKHSLLYEYFTVYN<br>ELTKVKYVTEGMRKPAFLSGEQKKAIVDLLFKTNRKVTVKQLKEDYFKKIECFDSVEISGVEDRFNA<br>SLGTYHDLLKIIKDKDFLDNEENEDILEDIVLTLTLFEDREMIEERLKTYAHLFDDKVMKQLKRRRYT<br>GWGRLSRKLINGIRDKQSGKTILDFLKSDGFANRNFMQLIHDDSLTFKEDIQKAQVGGGGSGGGGSG<br>GGGSCLSYETEILTVEYGLLPIGKIVEKRIECTVYSVDNNGNIYTQPVAQWHDRGEQEVFEYCLEDGS<br>LIRATKDHKFMTVDGQMLPIDEIFERELDLMRVDNLPNSGGSKRTADGSEFEPKKKRKV*

Intein-split PEmax p.714: p.NLS/C-intein/linker/n*Sp*Cas9$^{714-1368}$(H840A)/linker-NLS-linker/RT-dRnH/NLS
MKRTADGSEFESPKKKRKVIKIATRKYLGKQNVYDIGVERDHNFALKNGFIASNGGGGSGGGGSGG<br>GGSSGQGDSLHEHIANLAGSPAIKKGILQTVKVVDELVKVMGRHKPENIVIEMARENQTTQKGQKN<br>SRERMKRIEEGIKELGSQILKEHPVENTQLQNEKLYLYYLQNGRDMYVDQELDINRLSDYDVDAIVP<br>QSFLKDDSIDNKVLTRSDKNRGKSDNVPSEEVVKKMKNYWRQLLNAKLITQRKFDNLTKAERGGL<br>SELDKAGFIKRQLVETRQITKHVAQILDSRMNTKYDENDKLIREVKVITLKSKLVSDFRKDFQFYKV<br>REINNYHHAHDAYLNAVVGTALIKKYPKLESEFVYGDYKVYDVRKMIAKSEQEIGKATAKYFFYSN<br>IMNFFKTEITLANGEIRKRPLIETNGETGEIVWDKGRDFATVRKVLSMPQVNIVKKTEVQTGGFSKES<br>ILPKRNSDKLIARKKDWDPKKYGGFDSPTVAYSVLVVAKVEKGKSKKLKSVKELLGITIMERSSFEK<br>NPIDFLEAKGYKEVKKDLIIKLPKYSLFELENGRKRMLASAGELQKGNELALPSKYVNFLYLASHYE<br>KLKGSPEDNEQKQLFVEQHKHYLDEIIEQISEFSKRVILADANLDKVLSAYNKHRDKPIREQAENIIHL<br>FTLTNLGAPAAFKYFDTTIDRKRYTSTKEVLDATLIHQSITGLYETRIDLSQLGGDSGGSSGGSKRTA<br>DGSEFESPKKKRKVSGGSSGGSTLNIEDEYRLHETSKEPDVSLGSTWLSDFPQAWAETGGMGLAVR<br>QAPLIIPLKATSTPVSIKQYPMSQEARLGIKPHIQRLLDQGILVPCQSPWNTPLLPVKKPGTNDYRPVQ<br>DLREVNKRVEDIHPTVPNPYNLLSGLPPSHQWYTVLDLKDAFFCLRLHPTSQPLFAFEWRDPEMGIS<br>GQLTWTRLPQGFKNSPTLFNEALHRDLADFRIQHPDLILLQYVDDLLLAATSELDCQQGTRALLQTL<br>GNLGYRASAKKAQICQKQVKYLGYLLKEGQRWLTEARKETVMGQPTPKTPRQLREFLGKAGFCRL<br>FIPGFAEMAAPLYPLTKPGTLFNWGPDQQKAYQEIKQALLTAPALGLPDLTKPFELFVDEKQGYAKG<br>VLTQKLGPWRRPVAYLSKKLDPVAAGWPPCLRMVAAIAVLTKDAGKLTMGQPLVILAPHAVEALV<br>KQPPDRWLSNARMTHYQALLLDTDRVQFGPVVALNPATLLPSGGSKRTADGSEFESPKKKRKVGSG<br>PAAKRVKLD*
