# Supplementary material for: Prime editing of the β1 adrenoceptor in the brain restores physiological REM sleep in a mouse model of Alzheimer’s disease
Source: Nat Commun. 2025 Dec 9;16:10973. doi: 10.1038/s41467-025-65964-w (PMC12689628; doi:10.1038/s41467-025-65964-w)
Supplement: Supplementary file 2 — Reporting Summary [file 41467_2025_65964_MOESM2_ESM.pdf]

nature portfolio

Corresponding author(s): Gerald Schwank
Desiree Böck

Last updated by author(s): Oct 18, 2025

Reporting Summary

Nature Portfolio wishes to improve the reproducibility of the work that we publish. This form provides structure for consistency and transparency in reporting. For further information on Nature Portfolio policies, see our Editorial Policies and the Editorial Policy Checklist.

Statistics

For all statistical analyses, confirm that the following items are present in the figure legend, table legend, main text, or Methods section.

n/a	Confirmed	
☐	☒	The exact sample size (*n*) for each experimental group/condition, given as a discrete number and unit of measurement
☐	☒	A statement on whether measurements were taken from distinct samples or whether the same sample was measured repeatedly
☐	☒	The statistical test(s) used AND whether they are one- or two-sided<br>*Only common tests should be described solely by name; describe more complex techniques in the Methods section.*
☒	☐	A description of all covariates tested
☐	☒	A description of any assumptions or corrections, such as tests of normality and adjustment for multiple comparisons
☐	☒	A full description of the statistical parameters including central tendency (e.g. means) or other basic estimates (e.g. regression coefficient) AND variation (e.g. standard deviation) or associated estimates of uncertainty (e.g. confidence intervals)
☐	☒	For null hypothesis testing, the test statistic (e.g. *F*, *t*, *r*) with confidence intervals, effect sizes, degrees of freedom and *P* value noted<br>*Give P values as exact values whenever suitable.*
☒	☐	For Bayesian analysis, information on the choice of priors and Markov chain Monte Carlo settings
☒	☐	For hierarchical and complex designs, identification of the appropriate level for tests and full reporting of outcomes
☐	☒	Estimates of effect sizes (e.g. Cohen's *d*, Pearson's *r*), indicating how they were calculated

Our web collection on statistics for biologists contains articles on many of the points above.

Software and code

Policy information about availability of computer code

Data collection	Next-generation sequencing (NGS) data was collected and demultiplexed using the Illumina MiSeq Control software. RT-qPCR data were collected using a Lightcycler 480 system (Roche). FACS data was collected using LSR Fortessa (BD Biosciences) and the FACSDiva software version 8.0.1 (BD Biosciences). EM data were collected using an FEI Talos 120 kV transmission electron microscope (Thermo Fisher Scientific) equipped with a digital CMOS camera. LM data were collected using the Zeiss LSM 800 or the Zeiss AxioScan.Z1 slidescanner. Cleared whole-brain images were collected using a custom-made selective plane illumination microscope v.5 (www.mesospim.org). Behavior data was recorded using cameras (C270 HD Webcam, Logitech). Signal acquisition parameters for EEG/EMG recordings were set using the software SignalExpress NI 2015 controlled via LABVIEW.
Data analysis	NGS data were analyzed by CRISPResso2. CT values were calculated using the Lightcycler 480 software (Roche) and fold changes were calculated using the delta CT method. FACS data were analyzed using FlowJo 10.2. EM data were quantified using MAPS (Thermo Fisher Scientific) and Fiji. LM images were analyzed using Zeiss software Zen2 or Fiji (version 2.14.0). Whole-brain imaging data were analyzed using Fiji, ImageJ, ilastik, and elastix. Behavior data were tracked manually or automated using DeepLabCut 2.0.7. Infrared activity data were analyzed using ClockLab (Actimetrics). Vigilance states of EEG/EMG data were automatically scored using the automated scoring platform SPINDLE. All EEG/EMG analyses were performed in MATLAB (MathWorks R2020b) using published custome-made scripts. Statistical analyses were performed using GraphPad Prism (version 10.2.1) for MacOS.

For manuscripts utilizing custom algorithms or software that are central to the research but not yet described in published literature, software must be made available to editors and reviewers. We strongly encourage code deposition in a community repository (e.g. GitHub). See the Nature Portfolio guidelines for submitting code & software for further information.

Data

Policy information about availability of data

All manuscripts must include a data availability statement. This statement should provide the following information, where applicable:

- Accession codes, unique identifiers, or web links for publicly available datasets
- A description of any restrictions on data availability
- For clinical datasets or third party data, please ensure that the statement adheres to our policy

The main data supporting the results in the study are available within the paper and its Supplementary Information. Deep amplicon sequencing data is available at the SRA database under BioProject number PRJNA1168859 (https://www.ncbi.nlm.nih.gov/bioproject/PRJNA1168859/). EEG/EMG data has been deposited on Zenodo under accession number xxx (xxx).

Research involving human participants, their data, or biological material

Policy information about studies with human participants or human data. See also policy information about sex, gender (identity/presentation), and sexual orientation and race, ethnicity and racism.

Reporting on sex and gender	n/a
Reporting on race, ethnicity, or other socially relevant groupings	n/a
Population characteristics	n/a
Recruitment	n/a
Ethics oversight	n/a

Note that full information on the approval of the study protocol must also be provided in the manuscript.

Field-specific reporting

Please select the one below that is the best fit for your research. If you are not sure, read the appropriate sections before making your selection.

☒ Life sciences ☐ Behavioural & social sciences ☐ Ecological, evolutionary & environmental sciences

For a reference copy of the document with all sections, see nature.com/documents/nr-reporting-summary-flat.pdf

Life sciences study design

All studies must disclose on these points even when the disclosure is negative.

Sample size	Sample sizes were determined based on literature precedence for genome editing experiments (Villiger 2018, Gaudelli 2017, Koblan 2021, Rothgangl 2021, Böck 2022, Böck, 2024, Böck, 2024). Further power calculation was performed using the R 'pwr' package.
Data exclusions	No animals and no data were excluded.
Replication	All attempts at replication were successful. Findings were replicated as indicated in the figure legends.
Randomization	Newborn mice were assigned to the treated or untreated groups by litter. No covariates were controlled. Control and treatment groups for adult mice were randomized.
Blinding	For analysis of editing efficiencies, researchers were not blinded to group allocation. Blinding was not necessary here because the readout (editing efficiency) cannot be influenced by a biased researcher. All deep amplicon sequencing data was analyzed by an unblinded operator by using an automated script (CRISPResso2) with limited experimenter intervention. For performance and analysis of behavior and sleep experiments, researchers were blinded throughout the study.

Reporting for specific materials, systems and methods

We require information from authors about some types of materials, experimental systems and methods used in many studies. Here, indicate whether each material, system or method listed is relevant to your study. If you are not sure if a list item applies to your research, read the appropriate section before selecting a response.

Materials & experimental systems

n/a	Involved in the study
	☒ Antibodies
	☒ Eukaryotic cell lines
☒	☐ Palaeontology and archaeology
	☒ Animals and other organisms
☒	☐ Clinical data
☒	☐ Dual use research of concern
☒	☐ Plants

Methods

n/a	Involved in the study
☒	☐ ChIP-seq
	☒ Flow cytometry
☒	☐ MRI-based neuroimaging

Antibodies

Antibodies used

rat anti-CD11b mAb, FITC conjugated clone M1/70 (1:50; abcam catalogue number ab24874); mouse anti-Biotin mAb, PE conjugated clone Bio3-18E7 (1:50; Miltenyi Biotec, catalogue number 130-113-853); recombinant human anti-ACSA2 clone REA969 (1:50; Miltenyi Biotec, catalogue number 130-116-249), human anti-O4 mAB clone REA576 (1:50; Miltenyi Biotec, catalogue number 130-119-982); mouse anti-NeuN mAb (1:500; abcam catalogue number ab104224); chicken anti-Gfap (1:1500; abcam catalogue number ab4674); rabbit anti-Adrb1 pAb (1:1000; abcam catalogue number ab85037); mouse anti-actin beta mAb (1:2000, abcam catalogue number ab8226), rabbit anti-Iba1 pAb, clone 019-19741 (1:400; FUJIFILM Wako catalogue number 4987481428584).

Validation

rat anti-CD11b mAb: Validaton: https://www.abcam.com/fitc-cd11b-antibody-m170-ab24874.html; Validation reference: Meng Q, Zhang B, Zhang Y, Wang S, Zhu X. Human bone marrow mesenchymal stem cell-derived extracellular vesicles impede the progression of cervical cancer via the miR-144-3p/CEP55 pathway. J Cell Mol Med. 2021 Feb;25(4):1867-1883. doi: 10.1111/jcmm.15573. Epub 2021 Jan 8. PMID: 33417281; PMCID: PMC7882924.

mouse anti-Biotin mAb: Validation: https://www.miltenyibiotec.com/CH-en/products/biotin-antibody-bio3-18e7.html#conjugate=pe:size=30-tests-in-60-ul

recombinant human anti-ACSA2 mAb: Validation: https://www.miltenyibiotec.com/CH-en/products/acsa-2-antibody-anti-mouse-reafinity-rea969.html#conjugate=pe-vio-615:size=30-ug-in-200-ul; Validation reference: Sharma, K. et al. (2015) Cell type- and brain region-resolved mouse brain proteome. Nat. Neurosci. (12) 18: 1819 - 1831

human anti-O4 mAb: Validation: https://www.miltenyibiotec.com/CH-en/products/o4-antibody-anti-human-mouse-rat-reafinity-rea576.html#conjugate=apc:size=30-tests-in-60-ul; Validation reference: Bansal, R. et al. (1989) Multiple and novel specificities of monoclonal antibodies O1, O4, and R-mAb used in the analysis of oligodendrocyte development. J. Neurosci. Res. (4) 24: 548 - 557

mouse anti-NeuN, mAb: Validation: https://www.abcam.com/neun-antibody-1b7-neuronal-marker-ab104224.html; Validation reference: Li Q, Xiang YH, Liang XJ, Zhang Y, Zhao PP, Wang M, Bao XM, Zhu XB, Deng AC. Expression of G9a in Auditory Cortex Is Downregulated in a Rat Model of Age-Related Hearing Loss. J Mol Neurosci. 2021 Feb;71(2):409-418. doi: 10.1007/s12031-020-01663-z. Epub 2020 Jul 15. PMID: 32671696.

chicken anti-Gfap mAb: Validation: https://www.abcam.com/gfap-antibody-ab4674.html; Validation reference: Poon C, Pellow C, Hynynen K. Neutrophil recruitment and leukocyte response following focused ultrasound and microbubble mediated blood-brain barrier treatments. Theranostics. 2021 Jan 1;11(4):1655-1671. doi: 10.7150/thno.52710. PMID: 33408773; PMCID: PMC7778596.

rabbit anti-Adrb1 pAb: Validation: https://www.abcam.com/beta-1-adrenergic-receptor-antibody-ab85037.html; Validation reference: D'Adamo P, Horvat A, Gurgone A, Mignogna ML, Bianchi V, Masetti M, Ripamonti M, Taverna S, Velebit J, Malnar M, Muhič M, Fink K, Bachi A, Restuccia U, Belloli S, Moresco RM, Mercalli A, Piemonti L, Potokar M, Bobnar ST, Kreft M, Chowdhury HH, Stenovec M, Vardjan N, Zorec R. Inhibiting glycolysis rescues memory impairment in an intellectual disability Gdi1-null mouse. Metabolism. 2021 Mar;116:154463. doi: 10.1016/j.metabol.2020.154463. Epub 2020 Dec 10. PMID: 33309713; PMCID: PMC7871014.

mouse anti-actin beta mAb: Validation: https://www.abcam.com/beta-actin-antibody-mabcam-8226-loading-control-ab8226.html; Validation reference: Zhong Y, Lan J. Overexpression of Eukaryotic translation initiation factor 3D induces stem cell-like properties and metastasis in cervix cancer by activating FAK through inhibiting degradation of GRP78. Bioengineered. 2022 Jan;13(1):1952-1961. doi: 10.1080/21655979.2021.2024336. PMID: 35104170; PMCID: PMC8806159.

rabbit anti-Iba1 pAb: Validation: https://labchem-wako.fujifilm.com/us/product/detail/W01W0101-1974.html; Validation referece: Imai, Y., Ibata, I., Ito, D., Ohsawa, K. & Kohsaka, S.: Biochemical and biophysical research communications, 224(3), 855(1996).
A Novel Geneiba1 in the Major Histocompatibility Complex Class III Region Encoding an EF Hand Protein Expressed in a Monocytic Lineage

Eukaryotic cell lines

Policy information about cell lines and Sex and Gender in Research

Cell line source(s)

HEK293T (ATCC CRL-321), Hepa1-6 (ATCC CRL-1830) and Neuro2a (ATCC CCL-131)

Authentication	Cell lines were authenticated by the supplier using STR analysis.
Mycoplasma contamination	Cells were tested negative for mycoplasma.
Commonly misidentified lines (See ICLAC register)	No commonly misidentified cell lines were used.

Animals and other research organisms

Policy information about studies involving animals; ARRIVE guidelines recommended for reporting animal research, and Sex and Gender in Research

Laboratory animals	mus musculus (C57BL/6J; C57BL/6JRj; B6.Cg-Tg(Thy1-APPSw, Thy1-PSEN1*L166P)21Jckr)
Wild animals	The study did not involve wild animals.
Reporting on sex	Both female and male mice were used in this study. Sex was only considered during design and execution of EEG experiments since single-housing of female mice was not permitted on our animal license by the authorities.
Field-collected samples	The study did not involve samples collected from the field.
Ethics oversight	Animal experiments were performed in accordance with protocols approved by the Kantonales Veterinäramt Zürich and in compliance with the Swiss Animal Welfare Act and Protection Ordinance.

Note that full information on the approval of the study protocol must also be provided in the manuscript.

Plants

Seed stocks	n/a
Novel plant genotypes	n/a
Authentication	n/a

Flow Cytometry

Plots

Confirm that:

- [x] The axis labels state the marker and fluorochrome used (e.g. CD4-FITC).
- [x] The axis scales are clearly visible. Include numbers along axes only for bottom left plot of group (a 'group' is an analysis of identical markers).
- [x] All plots are contour plots with outliers or pseudocolor plots.
- [x] A numerical value for number of cells or percentage (with statistics) is provided.

Methodology

Sample preparation	Cells were isolated from freshly isolated mouse brains using the adult brain dissociation kit and magnetic activated cell sorting according to the manufacturer's instructions (Miltenyi Biotec). Isolaled cells were washed twice with phosphate-buffered saline (PBS) and resuspended in FACS Buffer (PBS supplemented with 2% FBS and 2 mM EDTA). Cell suspensions were filtered through 35 µm nylon mesh cell strainer snap caps (Corning) and kept on ice until analysis.
Instrument	LSR Fortessa (BD Biosciences)
Software	FACSDiva software version 8.0.1 (BD Biosciences)
Cell population abundance	For each sample, 20’000 events were counted.
Gating strategy	forward and side scatter area to select cells, side scatter height and side scatter area to select single cells; dead cells were identified by staining with eFluor780 (Thermo Fisher); living cells were then gated for cell type specitic markers (ACSA-2, O4, CD11b, Biotin)

- [x] Tick this box to confirm that a figure exemplifying the gating strategy is provided in the Supplementary Information.
