# Supplementary material for: Prime editing of the β1 adrenoceptor in the brain restores physiological REM sleep in a mouse model of Alzheimer’s disease
Source: Nat Commun. 2025 Dec 9;16:10973. doi: 10.1038/s41467-025-65964-w (PMC12689628; doi:10.1038/s41467-025-65964-w)
Supplement: Supplementary file 3 — Transparent Peer Review file [file 41467_2025_65964_MOESM3_ESM.pdf]

nature portfolio

Peer Review File

Prime editing of the β_1 adrenoceptor in the brain restores physiological REM sleep in a mouse model of Alzheimer's disease

Corresponding Author: Professor Gerald Schwank

This manuscript has been previously reviewed at another journal. This document only contains information relating to versions considered at Nature Communications.

This file contains all reviewer reports in order by version, followed by all author rebuttals in order by version.

Version 1:

Reviewer comments:

Reviewer #1

(Remarks to the Author)
With the additional revisions and transfer, the manuscript is appropriate for publication.

Reviewer #2

(Remarks to the Author)
I do not have any further concerns, and I believe that this revised manuscript is suitable for publication.

Reviewer #3

(Remarks to the Author)
In this revised version of the manuscript, the authors have corrected inconsistencies in the presentation of the sleep data and added further data and discussion to support a functional impact of prime editing on the noradrenergic system. The concerns raised in the previous revision regarding the sleep data have been satisfactorily addressed.

Open Access This Peer Review File is licensed under a Creative Commons Attribution 4.0 International License, which permits use, sharing, adaptation, distribution and reproduction in any medium or format, as long as you give appropriate credit to the original author(s) and the source, provide a link to the Creative Commons license, and indicate if changes were made.
In cases where reviewers are anonymous, credit should be given to 'Anonymous Referee' and the source.
The images or other third party material in this Peer Review File are included in the article's Creative Commons license, unless indicated otherwise in a credit line to the material. If material is not included in the article's Creative Commons license and your intended use is not permitted by statutory regulation or exceeds the permitted use, you will need to obtain permission directly from the copyright holder.
To view a copy of this license, visit https://creativecommons.org/licenses/by/4.0/
